# Chemopreventive and Anticancer Activity of Selected Triterpenoids in Melanoma

**DOI:** 10.3390/cancers17101625

**Published:** 2025-05-11

**Authors:** Natalia Dycha, Magdalena Michalak-Tomczyk, Jacek Jachuła, Estera Okoń, Agata Jarząb, Joanna Tokarczyk, Wojciech Koch, Katarzyna Gaweł-Bęben, Wirginia Kukula-Koch, Anna Wawruszak

**Affiliations:** 1 Department of Pharmacognosy with Medical Plants Garden, Medical University of Lublin, 1 Chodzki Str., 20-093 Lublin, Poland; dychanatalia98@gmail.com (N.D.); virginia.kukula@gmail.com (W.K.-K.); 2 Department of Physiology and Toxicology, The John Paul II Catholic University of Lublin, 1I Konstantynów Str., 20-708 Lublin, Poland; magdalena.michalak@kul.pl; 3 Department of Botany, Mycology and Ecology, Institute of Biological Sciences, Maria Curie-Skłodowska University, 19 Akademicka Str., 20-033 Lublin, Poland; jacek.jachula@mail.umcs.pl; 4 Department of Biochemistry and Molecular Biology, Medical University of Lublin, 1 Chodzki Str., 20-093 Lublin, Poland; estera.okon@umlub.pl (E.O.); agata.jarzab@umlub.pl (A.J.); 5 Department of Food and Nutrition, Medical University of Lublin, 4a Chodźki Str., 20-093 Lublin, Poland; joanna.tokarczyk@umlub.pl (J.T.); kochw@interia.pl (W.K.); 6 Department of Cosmetology, University of Information Technology and Management in Rzeszów, 2 Sucharskiego, 35-225 Rzeszów, Poland; kagawel@wsiz.edu.pl

**Keywords:** cancer, melanoma, terpenoids, triterpenoids, betulinic acid, oleanolic acid, glycyrrhetinic acid, ursolic acid, asiatic acid, madecassic acid

## Abstract

Melanoma is one of the most aggressive forms of skin cancer, characterized by high metastatic potential and resistance to conventional therapies. Natural compounds, particularly terpenoids, have gained attention for their chemopreventive potential and anticancer properties. These plant-derived compounds exhibit diverse biological activities, e.g., cell viability and proliferation inhibition, apoptosis induction, cell cycle regulation, and immune system modulation. The review evaluates the current state of the art on the chemopreventive and anticancer activity of lupane- (betulinic acid), oleanane- (oleanolic acid, β-amyrin, escin, hederagenin, glycyrrhetinic acid), and ursane-type (ursolic acid, asiatic acid, madecassic acid, α-amyrin) triterpenoids in melanoma, highlighting their mechanisms of action, therapeutic potential, and challenges in clinical application.

## Simple Summary

Terpenoids, a diverse group of natural compounds, have shown promising chemopreventive and anticancer effects in melanoma. These bioactive molecules target key pathways involved in tumor growth, metastasis, and drug resistance. Recent studies highlight their ability to induce apoptosis, inhibit cell proliferation, and modulate the immune response. Some terpenoids enhance the effectiveness of existing therapies, reducing side effects and improving patient outcomes. Ongoing research aims to identify the most potent triterpenoids and elucidate their mechanisms of action, providing a basis for future clinical investigations. This review presents an updated overview of triterpenoid-based strategies for melanoma treatment.

## 1. Introduction

Melanoma is the most aggressive type of skin cancer, originating from melanocytes. The incidence of melanoma has steadily increased in recent decades, particularly among individuals with fair skin, posing a significant challenge to global healthcare systems. Unlike many other solid tumors, melanoma frequently affects young and middle-aged individuals. Host-related risk factors, including the number of moles (both congenital and acquired), genetic predisposition, and family history, play a crucial role in melanoma development. Approximately 25% of cutaneous melanomas develop from preexisting moles [1]. The prognosis for patients with malignant melanoma varies by country; however, early detection has significantly reduced mortality rates [2].

**Figure 1:**
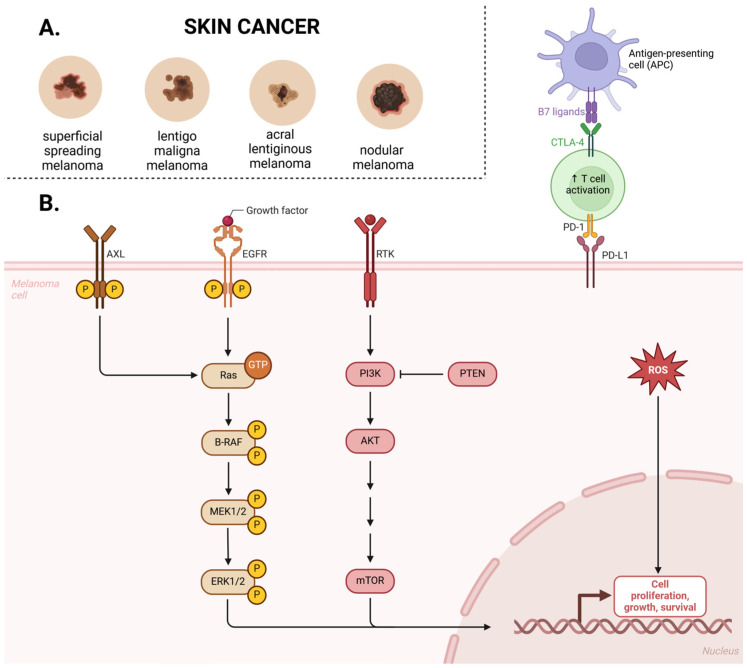
Four main subtypes of melanoma: superficial spreading melanoma, lentigo maligna melanoma, acral lentiginous melanoma and nodular melanoma (**A**). Signaling pathways are involved in melanoma progression. APC—antigen-presenting cell, CTLA-4—cytotoxic T-lymphocyte associated protein 4, EGFR—epidermal growth factor receptor, ERK 1/2—extracellular signal-regulated kinases 1/2, GTP—guanosine-5′-triphosphate, MEK 1/2—mitogen-activated protein kinase kinase 1/2, mTOR—mechanistic target of rapamycin kinase, PI3K—phosphoinositide 3-kinase, PD-1—programmed cell death protein 1, PD-L1—programmed cell death ligand 1, PTEN—phosphatase and tensin homolog, ROS—reactive form of oxygen, RTK—receptor tyrosine kinases (**B**).

Cutaneous melanoma is classified into four main subtypes: superficial spreading melanoma, lentigo maligna melanoma, acral lentiginous melanoma, and nodular melanoma (Figure 1A).

Superficial spreading melanoma accounts for approximately 70% of cases. This subtype primarily affects individuals with fair skin, typically appearing on the torso, and is strongly associated with excessive ultraviolet (UV) exposure. In individuals under the age of 40, melanoma most commonly presents as superficial spreading melanoma. In turn, lentigo maligna melanoma predominantly occurs in older individuals. Acral lentiginous melanoma primarily affects people with darker skin tones and typically appears as a dark lesion on the soles of the feet or palms. A specific form of this subtype, subungual melanoma, presents as dark streaks under the nails of the hands or feet. This is the rarest form of melanoma and the only one not associated with sun exposure [3].

In recent years, increasing attention has been directed toward the use of natural products as potential therapeutic agents in melanoma treatment. These compounds, derived from a diverse range of botanical, marine, and microbial sources, exhibit a broad spectrum of pharmacological activities, including antiproliferative, pro-apoptotic, anti-inflammatory, and antioxidant effects. Numerous preclinical studies have demonstrated that various classes of natural compounds—such as polyphenols, alkaloids, terpenoids, and flavonoids—can modulate key molecular pathways involved in melanoma progression, including those regulating cell cycle, apoptosis, angiogenesis, and metastasis. Their relative selectivity towards cancer cells and lower toxicity profiles compared to conventional chemotherapeutic agents further support their therapeutic potential [4].

Given the urgent need for more effective and safer therapeutic strategies against melanoma—a malignancy marked by its high metastatic propensity and resistance to standard interventions—this review aims to critically assess the current body of evidence regarding the chemopreventive and anticancer potential of selected lupane-, oleanane- and ursane-type triterpenoids. Specifically, the review focuses on betulinic acid within the lupane-type group, oleanolic acid, β-amyrin, escin, hederagenin, and glycyrrhetinic acid within the oleanane-type group, as well as ursolic acid, asiatic acid, madecassic acid, and α-amyrin within the ursane-type group. These naturally occurring pentacyclic triterpenoids are increasingly recognized for their multifaceted biological activities, including inhibition of melanoma cell viability and proliferation, induction of apoptosis, modulation of the cell cycle, and regulation of immune responses. By delineating their molecular mechanisms of action, evaluating their therapeutic potential, and discussing current limitations and translational challenges, this review provides an up-to-date synthesis that not only underscores the pharmacological relevance of these compounds but also offers novel insights into their prospective integration into melanoma prevention and treatment paradigms.

## 2. Genomic Changes in Melanoma

Over the past few decades, melanoma diagnostics have evolved from a primary reliance on histopathological evaluation, complemented by fundamental immunohistochemical markers, to increasingly sophisticated methodologies. A significant paradigm shift has occurred with the incorporation of high-sensitivity molecular assays, facilitating the early detection of premalignant lesions and enhancing diagnostic precision in melanoma screening programs [5].

In recent years, advancements in genomic analysis techniques have significantly enhanced the understanding of molecular alterations underlying melanoma carcinogenesis. Numerous mutated genes have been identified, impacting key signaling pathways that drive melanoma progression. Among the most critical pathways involved are the Ras/Raf/MAPK (mitogen-activated protein kinase) pathway, the AKT (protein kinase B) pathway, the cell cycle regulation pathway, the pigmentation-associated pathway, the p53 tumor suppressor pathway, and various epigenetic regulatory mechanisms (Figure 1B) [6].

The MAPK signaling pathway plays a pivotal role in melanoma pathogenesis, rendering it a critical therapeutic target [6]. Under physiological conditions, this pathway is tightly regulated, mediating signal transduction from extracellular stimuli to the nucleus via a sequential phosphorylation cascade. However, in melanoma, dysregulation frequently arises due to activating mutations in the *BRAF* and *RAS* genes, as well as other genetic and epigenetic alterations. This aberrant activation leads to sustained signaling, driving uncontrolled cell proliferation, enhanced migratory and invasive potential, metastatic dissemination, increased cell survival, and angiogenesis [7]. Activation of the MAPK pathway is initiated upon the binding of a growth factor to a receptor tyrosine kinase (RTK) at the cell surface, leading to the activation of Ras GTPase. The signal is subsequently propagated through the RAF kinase, followed by MAP2K1 (MEK1), and ultimately the ERK kinase cascade. Once phosphorylated, ERK translocates to the nucleus, where it modulates transcription factors that regulate genes involved in cell cycle progression and proliferation [1].

While the MAPK pathway is a central driver of melanoma progression, it does not fully encompass the diverse molecular mechanisms contributing to the disease. Emerging evidence suggests that additional signaling pathways play equally significant roles, with the phosphoinositide 3-kinase (PI3K)/AKT pathway being among the most extensively studied. PI3K is a heterodimer composed of a regulatory subunit (p85) and a catalytic subunit (p110). Activation of this pathway is initiated when the SH2 domain of p85 is engaged by receptor tyrosine kinases (RTKs) and their associated adaptor proteins, resulting in the recruitment of PI3K to the cell membrane. Additionally, the p110 subunit can be activated and translocated to the membrane following RAS activation. Upon activation, PI3K catalyzes the phosphorylation of phosphatidylinositol-4,5-bisphosphate (PIP2) at the 3′ hydroxyl position of the inositol ring, resulting in the generation of phosphatidylinositol-3,4,5-trisphosphate (PIP3), a critical secondary messenger in intracellular signaling. PIP3 serves as a docking site for serine/threonine kinases, including phosphoinositide-dependent kinase-1 (PDK1) and AKT, facilitating their activation. A critical negative regulator of the PI3K/AKT pathway is the tumor suppressor phosphatase and tensin homolog (PTEN), which dephosphorylates PIP3, thereby preventing AKT activation. Although the precise mechanisms governing PI3K pathway dysregulation in melanoma are not yet fully elucidated, the loss of PTEN expression or its functional inactivation is thought to contribute to aberrant signaling and tumor progression [8].

A critical molecular alteration in melanoma involves mutations in the GTPase *NRAS*, which occur in approximately 15–20% of cases. The most prevalent oncogenic mutations are localized to codons Q60/61 and G12/13, leading to constitutive activation of NRAS. As a key component of the Ras-RAF-MEK signaling cascade, NRAS belongs to the Ras protein family, which serves as an upstream regulator of RAF kinase activity. Given their functional interplay within this pathway, aberrant activation of NRAS and BRAF contributes to sustained mitogenic signaling and tumor progression [9].

The transforming growth factor-beta (TGFβ) signaling pathway represents a critical yet paradoxical regulator in melanoma pathogenesis, exhibiting both tumor-suppressive and tumor-promoting properties. In the early stages of carcinogenesis, TGFβ functions as a potent inhibitor of cellular proliferation in epithelial, endothelial, and hematopoietic lineages, thereby acting as a tumor suppressor. This cytostatic effect is mediated through the upregulation of cyclin-dependent kinase inhibitors, including p21, p27, and p15, which impede the G1-to-S-phase transition, effectively restraining uncontrolled cell cycle progression [10]. While certain studies have demonstrated that TGFβ suppresses tumor growth and invasive capacity in the murine B16F1 melanoma model, contrasting evidence indicates that excessive inhibition of TGFβ signaling—either through overexpression of the inhibitory Smad7 protein or pharmacological blockade of type I receptor activity—attenuates melanoma cell invasiveness and diminishes metalloproteinase secretion. These findings suggest that TGFβ may contribute to the pathogenesis of osteolytic bone lesions associated with malignant melanoma, underscoring its dualistic role in tumor progression [11].

In melanoma, the transcription factor p53 and its homologs, p63 and p73, exhibit a functional divergence from their canonical tumor-suppressive roles, failing to regulate apoptosis- and cell-cycle-related gene expression. This aberrant functionality implicates defective p53 activity in the facilitation of melanoma progression. Reduced p53 expression levels or mutational inactivation are associated with heightened tumor aggressiveness and therapeutic resistance. The intricate interplay among p53 isoforms contributes to its dysregulation, as these isoforms can form heterotetramers that impair transactivation capacity or assemble into inactive homotetramers that competitively bind DNA without eliciting transcriptional effects. Isoforms harboring a transactivation domain retain the capacity to activate p53 target genes, whereas dominant-negative isoforms lacking this domain exert inhibitory effects on the p53 family. Notably, p63 has been demonstrated to interact with p53 in melanoma, modulating its tumor-suppressive function. Furthermore, mutations in the *TP53* and *TP63* genes are frequently detected in melanomas harboring *BRAF* mutations, underscoring the interplay between p53 pathway disruption and oncogenic signaling in melanoma pathogenesis [12].

**Figure 2:**
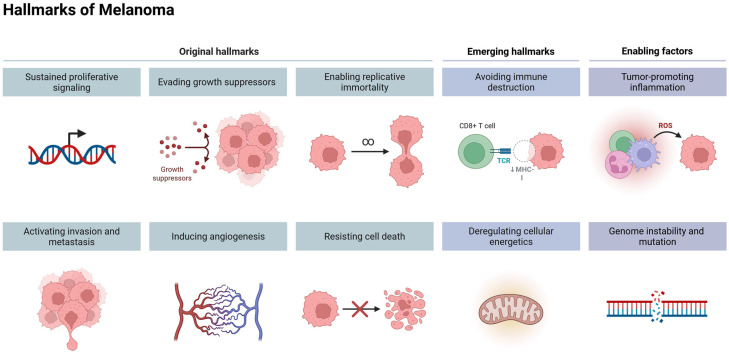
Melanoma hallmarks characterized by sustained proliferative signaling, evasion of growth suppressors, replicative immortality, avoidance of immune destruction, tumor-promoting inflammation, activation of invasion and metastasis, induction of angiogenesis, resistance to cell death, deregulated cellular energetics, and genome instability with high mutational burden.

The microphthalmia-associated transcription factor (MITF) serves as the master regulator of melanocyte development, function, and survival. In melanoma, MITF exhibits oncogenic properties, with gene amplification occurring in approximately 20% of cases, thereby facilitating tumor cell proliferation. MITF activation is governed by the MAPK and cAMP signaling pathways, orchestrating the transcription of key pigmentation-related enzymes, including tyrosinase (TYR), tyrosinase-related protein 1 (TYRP1), and dopachrome tautomerase (DCT). Within the melanoma microenvironment, aberrant ERK signaling—driven by oncogenic *BRAF* mutations—induces MITF degradation via a ubiquitin-mediated proteasomal pathway, contributing to dysregulated melanocytic lineage commitment and tumor progression (Figure 2) [1].

Current therapeutic options for melanoma remain limited, underscoring the urgent need for alternative treatment strategies. Emerging research increasingly focuses on the identification and development of novel therapeutic agents, particularly those derived from natural sources, which constitute a library of different organic scaffoldings that may become novel drug candidates. Bioactive compounds isolated from plants, marine organisms, and microorganisms have demonstrated potential in modulating critical molecular pathways implicated in melanoma progression, offering promising avenues for targeted intervention.

## 3. Terpenoids—Chemical Characteristics

Terpenoids (also called isoprenoids) represent the largest group of plant metabolites, with approximately 170,000 compounds with diverse linear and cyclic structures. The primary botanical families that serve as the major sources of terpenoids include Asteraceae, Lamiaceae, and Euphorbiaceae [13].

Terpenoids are classified as primary or secondary metabolites. Primary terpenoids, such as phytosterols, are essential components of plant cell membranes and contribute to fundamental processes like photosynthesis, growth, and development. In contrast, secondary terpenoids play specialized roles in plant defense against herbivores and pathogens, as well as in thermoregulation. Volatile terpenoids function as odor compounds that are capable of attracting pollinators. Specialized terpenoid production is often species- or tissue-specific and is not ubiquitous across all plants. Additionally, the biosynthesis of these compounds can be induced in response to environmental stress [14,15].

Terpenoids are derivatives of terpenes, where hydrocarbon compounds are synthesized from five-carbon isoprene units. Unlike terpenes, terpenoids contain additional heteroatoms, such as oxygen [16]. Terpenes are synthesized through cyclization processes catalyzed by terpene synthases. These compounds then undergo further modifications, including dehydrogenation, hydroxylation, glycosylation, or acylation, resulting in structurally diverse terpenoids. The biosynthesis of all terpenoids originates from two primary pathways: the classical mevalonate (MVA) pathway and the 2-C-methyl-D-erythritol 4-phosphate (MEP) pathway. These pathways generate isopentenyl diphosphate (IPP) and its allyl isomer, dimethylallyl diphosphate (DMAPP), which serve as universal precursors [15].

Terpenoids are classified based on the number of isoprene units into monoterpenoids, diterpenoids, triterpenoids, tetraterpenoids, and sesquiterpenoids [15]. The examples of compounds within each group and their natural sources are summarized in Table 1.

Plants containing essential oils are rich sources of compounds with strong aromatic properties, particularly from the monoterpenoid group, which can be classified into three subclasses: acyclic, monocyclic, and bicyclic structures [17]. Diterpenoids predominantly occur in cyclic forms and are categorized into several types, including the abietane, cuartene, cuaran, clerodane, labdane [26], tigliane, and daphne types [27]. Triterpenoids exist either as free, unbound compounds or as constituents of phytosterols and saponins. Triterpenoid saponins occur in glycosidic forms in numerous plant species and some marine organisms [28]. Tetracyclic dammarane-type structures represent the following subgroup of terpenoids. These compounds are present in sapogenins that can form triterpenoid saponins. In nature, the most widespread compounds of this type include ginsenosides, which are pharmacologically potent components characteristic of ginseng root [29]. Sesquiterpenoids also play an important role in the division of terpenoids. They are composed of three isoprene units and occur in nature in acyclic, cyclic, dimeric, or trimeric forms [30]. So far, more than 100 types of sesquiterpenoid skeletons have been identified [31].

**Figure 3:**
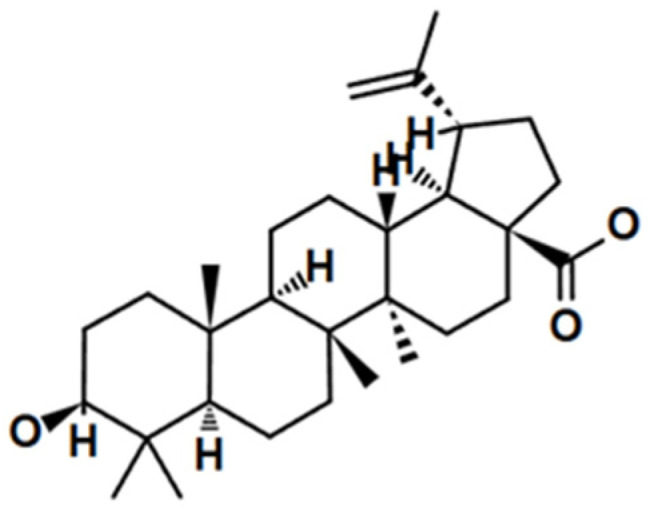
The chemical structure of betulinic acid.

This review manuscript aims to compile information on triterpenoids and their potential application in the treatment of melanoma. These are characterized by pentacyclic aglycones containing 30 carbon atoms. Among triterpene saponins, the lupane, oleanane, and ursane types have been distinguished. The former group, lupanes, has been isolated from a large number of spices, fruits, and medicinal plants [32]. The best studied metabolite is certainly betulinic acid (3β, hydroxy-lup-20(29)-en-28-oic acid) (Figure 3), which has drawn the attention of researchers due to its marked biological potential [33,34].

**Figure 4:**
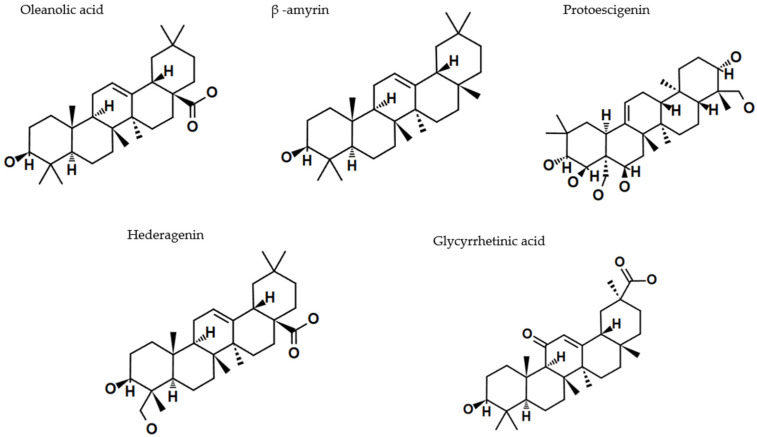
Chemical structures of the selected oleanane-type triterpenoids.

**Figure 5:**
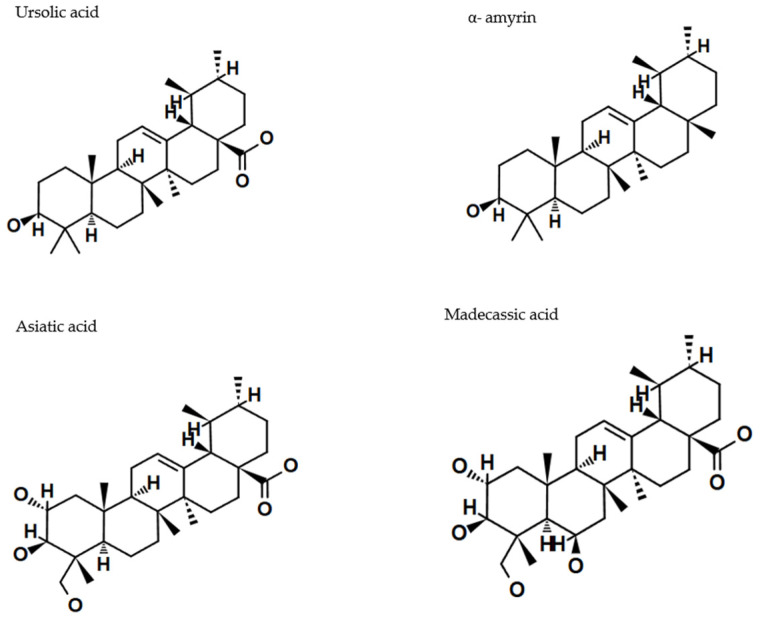
Chemical structures of selected ursane-type triterpenoids.

Ursolic acid (UA) (3β-hydroxyurs-12-en-28-oic acid) and oleanolic acid (OA) (3β-hydroxyolean-12-en-28-oic acid), whose name originates from its primary source, the European olive (*Olea europaea* L.), differ in the position of the methyl group. They represent two triterpenoid classes, namely the oleanane and ursane type, respectively. UA has an α-amyrin skeleton, whereas OA is based on a β-amyrin skeleton. Both compounds are widely distributed in medicinal and food plants, and because of this fact, they dominate the scientific publications on melanoma and other types of cancer [20]. Figure 4 and Figure 5 present selected structures of triterpenoids of the oleanane and ursane type, which will be further discussed in this paper.

**Figure 6:**
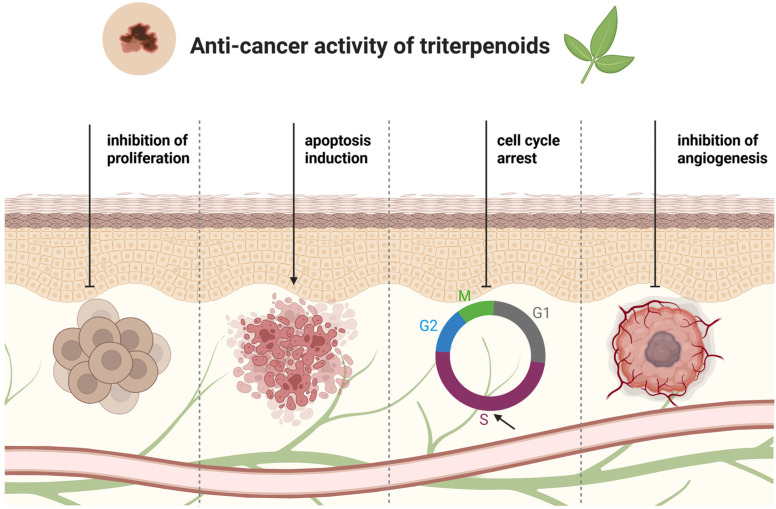
Anticancer activity of triterpenoids (inhibition of proliferation, apoptosis induction, cell cycle arrest, inhibition of angiogenesis) in melanoma.

Terpenoids, as a large group of compounds with highly diverse structures, hold significant potential for applications in medicine, as well as in the chemical, cosmetic, food, and biofuel industries. They are characterized by a broad spectrum of biological activities, including anti-inflammatory, antiprotozoal, antibacterial, and antiviral effects, along with cardioprotective and neuroprotective properties linked to their antioxidant activity [35,36,37]. They exhibit anticancer activity associated with antiproliferative, apoptotic, antiangiogenic, or antimetastatic effects (Figure 6) [26,38].

**Figure 7:**
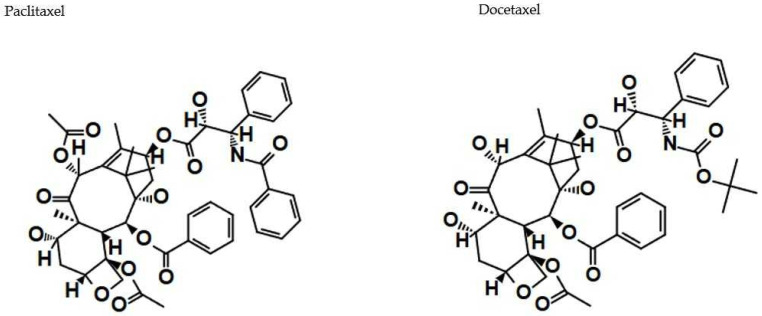
Chemical structures of paclitaxel and docetaxel.

As prominent examples of naturally occurring terpenoids of plant origin, two diterpenoids, paclitaxel and docetaxel, warrant mention. Both compounds have received FDA approval as effective anticancer agents and are considered first-line chemotherapeutics in the treatment of various malignancies, including ovarian cancer, breast cancer, lung cancer, and Kaposi’s sarcoma (Figure 7) [26].

Due to the low concentrations of these compounds in natural sources, the challenges associated with their isolation from plant extracts, and the complexities involved in their chemical synthesis, engineering strategies have been developed to produce these compounds in natural systems, such as microbiological and plant cultures, like rapidly growing strains of *Escherichia coli* and *Saccharomyces cerevisiae* [15].

## 4. Lupane-Type Triterpenoids in Melanoma Treatment—In Vitro Studies

### Betulinic Acid (BA)

Betulinic acid (BA) is a ubiquitous, lupane-type triterpenoid that is prominently featured in scientific publications concerning melanoma treatment, owing to its promising potential (see Table 2). One recent in vitro study investigated the efficacy of BA as a topical treatment for equine melanoma. The antiproliferative and cytotoxic effects of BA, as well as its ability to permeate isolated equine skin, were examined on primary equine melanoma cells and dermal fibroblasts. It was shown that BA inhibited both cell proliferation and metabolism in a time- and dose-dependent manner in melanoma cells and fibroblasts. Additionally, when 1% BA in “Basiscreme DAC” supplemented with 20% medium-chain triglycerides was applied to isolated equine skin, BA successfully reached high concentrations in the targeted skin layers. Antiproliferative and cytotoxic effects were observed as early as 5 h after application, although the affected cell population was still too small to determine IC_50_ values at this time. The effects became more pronounced with prolonged treatment, and the lowest IC_50_ values were recorded after 96 h of exposure. In conclusion, the significant percutaneous absorption of BA in equine skin, combined with its anticancer effects on melanoma cells, suggests that BA may have potential as an effective topical treatment for equine melanoma in vivo [39].

The other studies highlight the potential of *Rosmarinus officinalis* L. (RO) as a source of bioactive compounds capable of inhibiting AhR activation, which is linked to various inflammatory skin conditions. Previous research suggested that RO extracts effectively treated seborrheic dermatitis without adverse effects. Based on this, the effects of RO leaf extract and its key metabolites—40,7-*O*-dimethylapigenin, 7-*O*-methyl-epi-rosmanol, carnosol, carnosic acid, and BA—on AhR activity in human skin cells were examined. Then, it was revealed that a specially prepared RO extract functioned as an AhR antagonist against TCDD, PZ, IND, and FICZ, suggesting its potential in preventing or treating dioxin-related toxicity affecting multiple bodily systems, including the immune, endocrine, and nervous systems. Additionally, it was confirmed that the isolated compounds could block AhR activation by TCDD. However, their impact on AhR expression was not investigated, despite previous findings indicating that BA may enhance AhR expression through demethylation [40]. Furthermore, AhR has been linked to the development of skin cancers, including cutaneous squamous cell carcinoma. RO and carnosol have demonstrated tumor-suppressing properties, with carnosol application preventing tumor formation in experimental models. RO treatment also inhibited the binding of carcinogens like benzo[a]pyrene to skin cell DNA, reducing cancer risk. It has been suggested that RO and its constituents could serve as a novel pharmacological approach to managing AhR-related skin diseases and cancers [40].

Other recent research evaluated the anticancer properties of the BA derivative NVX-207 and the betulin derivative BBS on equine sarcoid (ES) cells, equine malignant melanoma cells, and fibroblasts. Both compounds exhibited significant antiproliferative and cytotoxic effects, with NVX-207 being the more potent agent. It was shown to effectively reduce cell viability and proliferation in both cell types by triggering the mitochondrial apoptotic pathway, activating caspase-3, -7, and -9, and inducing poly (ADP-ribose) polymerase cleavage. Additionally, increased subG1 cell populations were observed in various human cancer cell lines treated with BA and NVX-207. BBS, while also displaying antiproliferative effects, was less effective than NVX-207. However, it showed selectivity towards equine skin cancer cells over normal fibroblasts, as evidenced by Annexin V staining. It induced apoptosis in 82.1% of sarcoid cells after 48 h, whereas only 53.6% of fibroblasts and 28.2% of EMM cells were in the late apoptotic phase. Moreover, BBS inhibited human carbonic anhydrase isoenzymes I, II, and IX, with carbonic anhydrase IX being linked to tumor progression. Since malignant melanoma cells express this enzyme, combining its inhibition with proton pump inhibitors enhanced anticancer effects in vitro. Overall, NVX-207 demonstrated superior anticancer efficacy compared to BBS, showing strong skin penetration within 30 min of incubation. Despite promising in vitro results, further in vivo studies are necessary to determine its clinical potential for treating equine sarcoid and melanoma [41].

The therapeutic potential of BA and its derivatives was also explored, with a focus on their cytotoxic, antiproliferative, and pro-apoptotic effects on melanoma cells. Although BA has demonstrated antibacterial, antiviral, anti-inflammatory, and anticancer properties, its poor solubility and bioavailability limit its medical applications. To enhance its effectiveness, researchers developed several semi-synthetic derivatives incorporating an indole scaffold, known for its role in modulating G protein-coupled receptors (GPCRs) and potential therapeutic relevance in neurological diseases and cancer. The study assessed four BA derivatives (BA1–BA4) on murine melanoma (B164A5) and human melanoma (A375) cells, revealing a dose-dependent decrease in cell viability. Among them, BA1 exhibited the highest antiproliferative activity. Additionally, BA3 showed the strongest cytotoxic effects on B164A5 cells. The introduction of an indole group at the C2 position of BA significantly increased cytotoxicity, while modifications at the C28 position with glycine and glycylglycine residues (BA3 and BA2) further enhanced inhibitory activity. Higher concentrations (50–75 µM) resulted in negative cell viability, suggesting apoptotic induction, which was confirmed by Hoechst 33342 nuclear staining. Regarding toxicity towards normal cells, BA derivatives displayed selective cytotoxicity towards melanoma cells while causing only moderate effects on non-cancerous HaCaT cells. Concentrations up to 10 µM showed minimal impact on HaCaT cell viability, while higher doses (75 µM) reduced viability to approximately 70–80%, with BA4 exhibiting the least cytotoxicity. Additionally, BA itself reduced HaCaT cell viability by about 20% at concentrations above 10 µM. Furthermore, all tested compounds at 25 µM or higher led to significant LDH release in murine melanoma cells after 72 h, indicating membrane damage and cell death. Overall, the enhanced anticancer potential of BI derivatives, particularly BA1, BA2, and BA3, were revealed, while maintaining moderate toxicity towards normal cells [41]. Further, the abovementioned BA derivatives (BA1-BA4) were tested on human melanoma A375 cells. Compounds BA1, BA2, and BA3 exhibited enhanced (2–3 times) anti-proliferative activity compared to BA. The LDH leakage test revealed that the mechanism lying behind cell death is associated with cell membrane function disruption. Additionally, BA and its derivatives showed the ability to decrease cell migration rates in a dose-dependent manner, reaching the level of 2.1% at a concentration of 50 µM in the case of BA [42].

Another study tested the efficacy of BA formulation with gold nanoparticles (BA-GNP) against human melanoma cell line RPMI-7951 and normal human keratinocytes (HaCaT). BA-GNP significantly reduced cell viability in melanoma cells at concentrations of 25 and 50 µM (75.1% and 63.4% of viable cells, respectively), while toxicity against normal keratinocytes was observed only at the concentration of 50 µM (86.9% viable cells). Apoptotic changes (nuclear shrinkage and fragmentation) in melanoma cells were most visible at the concentration of 50 µM. The mitochondrial-targeted action of BA-GNP was confirmed by the decreased mitochondrial respiration rates. A HET-CAM irritation assay also confirmed the biocompatibility of gold nanoparticles with mucosal tissues (Table 2) [43].

To sum up, recent in vitro studies have confirmed the efficacy of BA in the treatment of melanoma, demonstrating its antiproliferative and cytotoxic effects on primary equine melanoma cells and dermal fibroblasts. The studies revealed that BA inhibits both cell proliferation and metabolism in a time- and dose-dependent manner. Notably, BA was able to penetrate isolated equine skin effectively, achieving high concentrations in targeted skin layers after application. Antiproliferative and cytotoxic effects were observed as early as 5 h post-application, with more pronounced effects at 96 h. The substantial percutaneous absorption of BA, combined with its anticancer effects on melanoma cells, suggests its potential as an effective topical treatment for equine melanoma in vivo.

Additionally, studies on BA derivatives have highlighted their enhanced anticancer properties. For instance, derivatives demonstrated selective cytotoxicity towards melanoma cells while minimally affecting normal cells. Moreover, formulations combining BA with gold nanoparticles showed significant reductions in melanoma cell viability while maintaining biocompatibility with normal keratinocytes. These findings underscore the therapeutic promise of BA and its derivatives in melanoma treatment, warranting further investigation into their clinical applications.

**Table 2:** Betulinic acid (BA) activity in melanoma in vitro treatment.

Type	Cell Line	Effect	Concentration/IC_50_ Values	Reference
BA	Self-generated primary equine dermal fibroblasts PriFi1 and PriFi2 and previously isolated primary equine melanoma cells (MelDuWi)	↓ cell proliferation ↑cell cytotoxicity	MelDuWi: IC_50_ = 34.6 µM, PriFi1: IC_50_ = 20.4 µM, PriFi2: IC_50_ = 24.8 µM	[39]
BA	Human melanoma cell lines (A375, SK-MEL28, FM55P, and FM55M2), normal human keratinocytes (HaCaT)	↓ cell viability	1–40 µM, A375: IC_50_ = 15.94 µM, SK-MEL28: IC_50_ = 2.21 µM, FM55P: IC_50_ = 5.62 µM, FM55M2: IC_50_ = 4.08 µM	[44]
BA	Human melanoma cell line (IGR1), normal human keratinocytes (HaCaT)	↑ apoptosis DNA fragmentation	IGR1: IC_50_ = 1.3 µg/mL (2.85 µM, respectively); normal cells: IC_50_ = 5 µg/mL (10.95 µM, respectively)	[45]
Rosemary extract containing BA	Human keratinocytes (SIK 28) cells	Inhibition of TCDD-induction of AhR-dependent reporter gene expression, inhibition of TCDD-stimulated AhR transformation and DNA binding	10–100 µM	[40]
BBS and BA derivatives	MelDuW, PriFri2, and sRGO2	↓ cell proliferation ↑ cell cytotoxicity	1–100 µM	[40]
BA and BA derivatives: *N*-(2,3-indolo-betulinoyl)diglycylglycine (BA1), *N*-(2,3-indolo-betulinoyl)glycylglycine (BA2), and *N*-(2,3-indolo-betulinoyl)glycine (BA3), 2,3-indolo-betulinic acid (BA4)	Murine melanoma cells (B164A5)	LDH leakage, cell membrane disruption, altered cell nuclei morphology	B164A5: 1–40 µM; BA: IC_50_ = 21.14 µM, BA1: IC_50_ = 10.34 µM, BA2: IC_50_ = 9.15 µM, BA3: IC_50_ = 8.11 µM, BA4: IC_50_ = 17.62 µM	[41]
BA and BA derivatives: *N*-(2,3-indolo-betulinoyl)diglycylglycine (BA1), *N*-(2,3-indolo-betulinoyl)glycylglycine (BA2), and *N*-(2,3-indolo-betulinoyl)glycine (BA3), 2,3-indolo-betulinic acid (BA4)	Human melanoma cell lines (A375)	↓ cell viability, LDH leakage, ↓ cell migration	1, 10, 25, 50, and 75 µM; BA: IC_50_ = 19.2 µM, BA1: IC_50_ = 5.7 µM, BA2: IC_50_ = 13.7 µM, BA3: IC_50_ = 10.0 µM, BA4: IC_50_ = 19.6 µM	[42]
BA-GNP	Human melanoma cell lines (RPMI-7951)	↓ cell viability ↓ Bcl-2 production ↑Bax production ↓ mitochondrial respiration, cell shrinkage and deformation, nuclear condensation, shrinkage and fragmentation	10, 25, and 50 µM	[43]

BA—betulinic acid, BBS—betulinyl-bis-sulfamate, LDH—lactate dehydrogenase, ↓—decreased/alleviated, ↑—increased/induced.

## 5. Oleanane-Type Triterpenoids in Melanoma Treatment—In Vitro Studies

### 5.1. Oleanolic Acid (OA)

Oleanolic acid was also found to be a promising candidate for the treatment of melanoma (see Table 3). In the former studies, OA induced a concentration- and time-dependent decrease in cell viability in studies on A375 melanoma cells. Wright–Giemsa staining revealed morphological changes in cells treated with OA for 24 h. At higher concentrations (>50 µM), typical apoptotic features were observed, including loss of cell–cell contacts, membrane blebbing, and membrane damage. Furthermore, the apoptotic effects of OA were evaluated using a cellular DNA fragmentation assay. BrdU-labeled DNA fragments, generated following OA pre-treatment, were quantified by measuring absorbance at 450 nm. A concentration-dependent increase in DNA fragmentation was observed in A375 cells, confirming the dose-dependent pro-apoptotic activity of OA [46]. In another study using the same cell line, the authors observed a dose-dependent reduction in melanoma cell viability after 24 h of OA exposure, with an IC_50_ value of approximately 41 μM, while normal skin keratinocyte cells showed minimal cell death. The cytotoxicity of OA was also associated with apoptosis, as confirmed by the induction of inter-nucleosomal DNA fragmentation detected by increased DAPI staining intensity, as well as PARP degradation following OA treatment. Additionally, analysis of cell cycle progression demonstrated that OA exposure led to increased accumulation of cells in the G0/G1 phase of the cell cycle. Immunoblot and densitometric analyses revealed downregulation of downstream effectors of EGFR, such as pAkt, pSTAT5, and Erk1/2. At the molecular level, OA upregulated the expression of pro-apoptotic proteins such as p53, cytochrome c, and Bax, while downregulating the anti-apoptotic protein Bcl-2. Furthermore, flow cytometry analysis after OA exposure revealed dose-dependent changes in mitochondrial membrane potential (MMP), which are associated with the release of cytochrome c into the cytosol. Moreover, it was observed that OA treatment of A375 cells led to the activation of caspase-3, as confirmed by immunoblot assays. These findings clearly indicate that OA induces apoptosis through the intrinsic mitochondrial pathway [47]. Similarly, in the study by Woo et al., OA was shown to induce apoptosis in A375SM and A375P melanoma cells by regulating the expression of apoptosis-related proteins. Additionally, the analysis of NF-κB pathway-related proteins in OA-treated melanoma cells revealed an increase in p-IKKαβ expression and a decrease in p-IκBα and p-NF-κB levels in A375SM cells compared to the control group. Furthermore, treatment with 80 μM OA led to elevated NF-κB levels, likely due to complex bio-signaling mechanisms that require further investigation. In contrast, OA treatment in A375P cells resulted in decreased expression of p-IKKαβ, p-IκBα, and p-NF-κB. These findings suggest that OA induces apoptosis in both A375SM and A375P melanoma cells through the NF-κB pathway [48].

The molecular mechanisms through which OA promotes wound healing were investigated, specifically by enhancing cell migration. The research focused on two epithelial cell lines, showing that OA significantly enhances cell migration in scratch assays. This migration effect was linked to the activation of key proteins like c-Jun and ERK1/2, both of which are critical for cell movement. Additionally, gene expression analysis revealed a profile indicative of a pro-migratory state and enhanced ability for extracellular matrix (ECM) remodeling, supporting the observed wound healing effects of OA. Interestingly, OA’s migratory effects were still evident at low concentrations, even when proliferation was minimized, such as in experiments where cell proliferation was inhibited by mitomycin C. In these conditions, OA still enhanced migration, suggesting that its effects on migration are somewhat independent of cell proliferation. However, when serum was added to the culture, OA’s migratory benefits were less pronounced because serum factors not only enhanced migration but also increased cell proliferation, overshadowing OA’s contribution. It was revealed that OA’s effects on migration are partially mediated through the activation of the EGFR. When EGFR was blocked, OA’s migratory enhancement was prevented. Moreover, the involvement of MAP kinases, specifically JNK1/2 and ERK1/2, was crucial for OA’s effects. OA activated both pathways, and inhibiting them with specific blockers (JNKi and MEKi) impaired the migration, underscoring their importance in the migratory process. However, the inhibition of p38 MAPK did not impact OA-induced migration, suggesting that p38 might not play a significant role in this context. Gene expression analysis further confirmed OA’s influence on key migratory genes. Notably, OA upregulated c-Jun and CDKN1A (encoding the protein p21), which regulate cell cycle arrest. This regulation may explain the slight anti-proliferative effects of OA observed in certain conditions. Additionally, OA also induced the expression of *SNAI2* and *FOXO1*, transcription factors involved in cell migration and epithelial plasticity, supporting its pro-migratory effects. In conclusion, this study provides molecular evidence for the wound-healing effects of OA, linking them to the activation of key signaling pathways and gene expression changes. These findings validate the traditional use of OA in wound healing and offer new insights that could guide future research and innovations in regenerative medicine [49].

The protective effects of OA against oxidative stress were shown in keratinocytes, particularly through its influence on the inducible nitric oxide synthase (iNOS) expression, nitric oxide (NO) production, and the Nrf2 signaling pathway. Exposure to the tert-butyl hydroperoxide (tBHP) significantly increases iNOS expression, leading to elevating NO levels, a phenomenon closely linked to immune and inflammatory responses in the skin. Dysregulated iNOS activity and excessive NO production are associated with various skin disorders, making OA’s ability to reduce these effects particularly noteworthy. It was suggested that OA not only counteracts ROS accumulation but also downregulates iNOS expression, thereby modulating NO levels. This mechanism highlights OA’s potential as a therapeutic agent for oxidative stress-related skin conditions. To further elucidate OA’s cytoprotective role, its impact on the Nrf2 pathway, which orchestrates the cellular defense against oxidative damage, was explored. Under normal conditions, Nrf2 remains at low levels, but oxidative stimuli, such as tBHP, trigger its activation and nuclear translocation, leading to the upregulation of antioxidant genes like heme-oxygenase 1 (*HO-1*). It was confirmed that tBHP exposure stimulates Nrf2 accumulation and enhances *HO-1* expression, supporting the established role of this pathway in restoring redox balance and mitigating ROS toxicity. An additional layer of regulation involves the MAPK signaling pathway, which was also activated in response to tBHP-induced oxidative stress. This aligns with existing research highlighting MAPK’s involvement in cellular stress responses, including its role in Nrf2 induction. The interplay between these pathways appears crucial for cellular adaptation to oxidative insults. However, pre-treatment with OA altered this response. Cells exposed to OA before tBHP stimulation did not exhibit the expected activation of the Nrf2 pathway. Instead, their Nrf2 and *HO-1* expression levels remained comparable to untreated control cells, suggesting that OA prevents oxidative stress from occurring rather than triggering an adaptive response. The absence of significant ROS accumulation in OA-treated cells supports its direct antioxidant function and stabilizing effect on redox homeostasis. It is thus suggested that OA mitigates oxidative damage by preemptively neutralizing ROS rather than relying on secondary defense mechanisms. Its ability to reduce iNOS expression and excessive NO production, combined with its influence on the Nrf2 and MAPK pathways, underscores its potential as a protective agent against oxidative stress-related skin pathologies. By stabilizing cellular redox balance and preventing ROS accumulation, OA emerges as a promising candidate for future therapeutic applications in dermatology [50].

Other studies explored the cytotoxic effects of OA derivatives across a range of cancer cell lines. The findings indicated that the effects observed were largely dependent on the specific cell line’s susceptibility. Nevertheless, the research also highlighted certain patterns in the relationship between the chemical structure and biological activity. A key structural feature influencing the cytotoxic potential of the OA derivatives was found to be the free carboxyl group at position C-17 (28-COOH) in the OA structure. When this carboxyl group was blocked by attaching a sugar chain, the cytotoxic activity was significantly diminished or entirely abolished (Table 3) [51].

As proved above, OA has emerged as a promising candidate for melanoma treatment, demonstrating significant cytotoxic effects on A375 melanoma cells. Studies indicate that OA induces a concentration- and time-dependent decrease in cell viability, with apoptotic characteristics evident at concentrations exceeding 50 µM. Morphological assessments revealed typical apoptotic features such as loss of cell–cell contacts, membrane blebbing, and damage. Furthermore, DNA fragmentation assays confirmed OA’s pro-apoptotic activity, with increased DNA fragmentation correlating with higher concentrations of OA. Mechanistically, OA triggered apoptosis through the intrinsic mitochondrial pathway by downregulating anti-apoptotic proteins like Bcl-2 and upregulating pro-apoptotic proteins such as p53 and Bax. This process is accompanied by a decrease in the phosphorylation of key signaling molecules downstream of the epidermal growth factor receptor (EGFR), including pAkt and Erk1/2. OA also altered cell cycle progression, leading to increased accumulation of cells in the G0/G1 phase and enhanced cell migration and wound healing through the activation of signaling pathways involving c-Jun and ERK1/2, while also exhibiting protective effects against oxidative stress by modulating nitric oxide production and the Nrf2 signaling pathway. Overall, OA’s multifaceted mechanisms underscore its potential as an effective therapeutic agent in melanoma and other skin conditions.

**Table 3:** Oleanane-type terpenoids in melanoma in vitro treatment.

Type	Cell Line	Effect	Concentration/IC_50_ Values	Reference
OA	Human keratinocytes (HaCaT) cells exposed to the pro-oxidative agent tBHP	↑ cell viability, ↓ intracellular ROS levels, ↓ inducible iNOS	1.25 μg/mL (2.74 µM, respectively)	[50]
OA	Human melanoma cell lines (HTB140, A375, WM793), human keratinocytes (HaCaT)	↑cell cytotoxicity	0.5–100 μg/mL (1.1–219 μM, respectively)	[51]
OA	Human melanoma cell line (A375)	↓ cell viability, ↑ cell wall disruption, ↑ apoptosis	IC_50_ = 277.5 μM	[46]
OA	Human melanoma cell line (A375)	↓ cell viability, ↑ apoptosis, ↑ G0/G1 proliferation arrest, ↑ inter-nucleosomal fragmentation, up-regulation of Bax, down-regulation of Bcl-2, ↑ cytochrome c release to cytosol	IC_50_ = 40.70 μM	[47]
OA	Human melanoma cell lines (A375SM, A375P)	↓ cell viability, ↑ expression of the apoptotic proteins (PARP, Bax), ↓ expression of Bcl-2, ↓ expression of p–NF–κB and *p*-IκBα	20–100 μM	[48]
OA; OA derivative (3-*O*-succinyl-28-*O*-benzyl oleanolate)	Mouse melanoma cell line (B16-F10)	↓ cell proliferation, ↑ apoptosis, ↑ G_0_/G_1_ cell-cycle arrest, chromatin condensation and fragmentation, loss of membrane asymmetry, cell shrinkage	IC_50_ = 46.2 μg/mL (101.18 μM, respectively); IC_50_ = 15.3 μg/mL (22. 8 μM, respectively)	[52]
OA derivative (tryptamine amide of (3*β*)-3-(acetyloxy)olean-12-en-28-oic acid)	Human melanoma cell line (G-361)	↑ cytotoxicity, ↑ apoptosis, ↑ accumulation of the S-phase cells, ↓ G_2_/M phase cells	IC_50_ = 9.0 µM	[53]
*β*-amyrin	Human melanoma cell line (A375)	↓ cell proliferation	IC_50_ = 0.48 µM	[54]
Escin	Human melanoma cell line (CHL-1)	↑ apoptosis, ↑ cell cytotoxicity, ↑ ROS generation	IC50 = 6 μg/mL (13.57 μM, respectively)	[55]
Escin	Human melanoma cell lines (B16F10 and SK-MEL5)	↑ inhibitors of metalloproteinases 1 and 2 (TIMP-1 and TIMP-2) expression, ↓ phosphorylated extracellular signal-regulated kinase (p-ERK), ↓ expression of nuclear factor-kappa B (NF-κB) and its inhibitor, IκB	20 µM	[52]
Hederagenin	Human melanoma cell line (SK-MEL-2)	↓ cell viability	IC_50_ = 27.67 µM	[56]
Hederagenin; hederagenin 3-*O*-[*α*-L-rhamnopyranosyl-(1→2)-*α*-L-arabinopyranoside]; hederagenin 3-*O*-[*β*-D-glucopyranosyl-(1→3)-*α*-L-arabinopyranoside]	Human melanoma cell line (SK-MEL-2)	↓ cell viability	IC_50_ = 22.0 µg/mL (17.98 µM respectively); IC_50_ = 2.3 µg/mL; IC_50_ = 7.0 µg/mL	[57]
δ-hederin; Glc3-O-hederagenin; hederacolchicosid A; α-hederin	Malignant melanoma cell line (M4 Beu)	↓ cell viability	IC_50_ = ca. 30 µM; IC_50_ = ca. 35 µM; IC_50_ = ca. 10 µM; IC_50_ = ca. 25 µM	[58]
3-*O*-*β*-D-glucopyranosyl-(1 → 2)-*β*-D-galactopyranosyl hederagenin 28-*O*-*α*-L-rhamnopyranosyl-(1 → 4)-*β*-D-glucopyranosyl-(1 → 6)-*β*-D-glucopyranoside	Human melanoma cell line (A375)	↓ cell proliferation	IC_50_ = 21.4 µg/mL	[59]
GA derivative (GPD-12)	Human melanoma cell line (A375) and murine cell line (B16F10)	↑cell cytotoxicity, ↑ nuclear fragmentation, ↑ expression of apoptosis related protein, e.g., caspase-3 and caspase-9, ↑ Bax to Bcl2 ratio	5–100 μM	[60]
GA derivative (3-*O*-prenyl glycyrrhetinic acid)	Human melanoma cell lines (A375, SKMEL-28), murine melanoma cell line (B16F10), and normal human keratinocytes (HaCaT)	↓ activity of MAPK signaling pathway, ↓ AKT survival signaling pathway, ↑ expression of p-mTOR, ↑ ER stress in cells, ROS generation	16, 27, 33.5 or 50 μM	[61]

OA—oleanolic acid, tBHP—tert-butyl hydroperoxide, ROS—reactive form of oxygen; iNOS—inducible nitric oxide synthase; p-IκBα—phospho-inhibitor of nuclear factor-κBα, p–NF–κB—phospho-nuclear factor-κB, NF-κB—nuclear factor-kappa B, GA—glycyrrhetinic acid, MAPK—mitogen-activated protein kinase, ↓—decreased/alleviated, ↑—increased/induced.

### 5.2. β-Amyrin

Several active compounds, including triterpenes, were identified by implementation of the HPLC-MS analysis of the methanolic extract from *T. alternans* aerial parts. Chromatographic separation of the lipophilic fraction led to the isolation of six triterpenes (T1–T6) that were further identified by NMR spectroscopy, with T2 and T3 being recognized as α- and β-amyrin. Cytotoxicity tests demonstrated that the isolated triterpenes exhibited significant anticancer activity, surpassing that of BA across all tested cancer cell lines, including melanoma (Table 3) [54].

### 5.3. Escin

Escin, a triterpenoid saponin derived from plants and present in the highest quantities in different species of chestnut, is a bioactive natural complex of saponins with a broad therapeutic potential. Initially recognized for its potent anti-inflammatory and anti-edematous properties, escin has recently gained attention for its emerging anticancer activities (Table 3). Over the past two decades, studies have demonstrated its efficacy in combination with approved anticancer agents, enhancing their apoptotic, anti-metastatic, and anti-angiogenic effects through synergistic interactions and improved bioavailability [62]. Escin exhibited a dose-dependent reduction in CHL-1 melanoma cell viability within 24 h of exposure, demonstrating high cytotoxicity at lower concentrations (IC_50_ = 6 μg/mL). Mechanistically, escin inactivated Bcl-2 signaling and induced apoptosis by promoting ROS generation. Additionally, it triggered morphological and ultrastructural alterations consistent with pro-apoptotic activity [55]. Another study demonstrated that *β*-escin at 20µM inhibits cell migration and motility in B16F10 and SK-MEL5 melanoma cells in a dose-dependent manner. RT-PCR and Western blot analyses revealed that *β*-escin upregulated the tissue inhibitors of metalloproteinase 1 and 2 (TIMP-1 and TIMP-2) expression while significantly downregulating phosphorylated extracellular signal-regulated kinase (p-ERK). Additionally, *β*-escin suppressed the expression of nuclear factor-kappa B (NF-κB) and its inhibitor, IκB. Collectively, these findings suggest that *β*-escin possesses anti-metastatic and anti-angiogenic potential, providing the first evidence of NF-κB/IκB signaling involvement in *β*-escin-induced antitumor effects [63].

### 5.4. Hederagenin

Hederagenin is an aglycone that is widely distributed among medicinal plants (especially in *Hedera helix*). It is a sapogenin, which constitutes a scaffolding of a large number of saponins in their glycosylated forms [64]. Hederagenin glycosides exhibit more potent cytotoxic effects than the sapogenin itself. For example, in the sulforhodamine B assay, the cytotoxic effect on human melanoma SK-MEL-2 line is much stronger when using hederagenin 3-*O*-[*α*-L-rhamnopyranosyl-(1→2)-*α*-L-arabinopyranoside] (IC_50_ = 2.3 µg/mL) and hederagenin 3-*O*-[*β*-D-glucopyranosyl-(1→3)-*α*-L-arabinopyranoside (IC_50_ = 7.0 µg/mL) compared to hederagenin (IC_50_ = 22.0 µg/mL) (Table 3) [57]. It is also the sugar attachment that shapes the cytotoxic activities of hederagenin glycosides. By the comparison of δ-hederin (IC_50_ = ca. 30 µM) and Glc3-O-hederagenin (IC_50_ = ca. 35 µM) effects on malignant melanoma M4 Beu cells, it was shown that the replacement of arabinose by glucose in a hederagenin monodesmoside decreases its cytotoxicity. Moreover, the stronger anti-proliferative properties of hederacolchicosid A (IC_50_ = ca. 10 µM) compared to α-hederin (IC_50_ = ca. 25 µM) confirm that the effect of hederagenin monodesmosides is improved by the presence of the second sugar attachment [58].

### 5.5. Glycyrrhetinic Acid (GA)

Several studies indicate that GA selectively targets tumor cells without harming normal cells and exhibits a stronger anticancer effect than some existing treatments, though its use as a cytotoxic agent is limited by its high hydrophobicity and poor solubility in blood serum. Recent research has focused on structural modifications to improve GA’s bioavailability and effectiveness [65]. Thus, through various chemical modifications, increased cytotoxic activity (with IC_50_: ≤10 µM) has been observed against skin cancer cells, including lines such as A375, A518A2, A253, and SK-MEL. These modifications include the C-3(OH) group in ring A and the C_30_-CO_2_H group in ring E, which are key structural features that make GA an appealing scaffold for medicinal chemistry. Moreover, compounds with cyanoenone groups in ring A or those containing amino groups or nitrogen-based heterocycles exhibit heightened cytotoxicity [66,67,68,69].

Recently, GA derivatives (developed using the established Fischer indole synthesis method) were screened against B16F10 and A375 melanoma cells. Among all the derivatives, GPD-12 exhibited a significant selective decrease in cell viability for both human and mouse melanoma cell lines, with IC_50_ values of 13.38 μM and 15.2 μM, respectively. It demonstrated lower toxicity to normal skin cells, with an IC_50_ of 65.52 μM. H_2_DCF-DA fluorescent imaging revealed that GPD-12 caused a dose-dependent increase in ROS levels in A375 melanoma cells after 6 h of treatment compared to the control cells (HDF). This, in turn, induces oxidative stress, which may lead to cell death through apoptosis. Additionally, based on DAPI staining, the presence of pits and grooves was observed in GPD-12-treated cells, indicating that GPD-12 targets the nucleus and causes DNA damage in melanoma cells. Western blotting revealed that this GA derivative significantly increased the expression of caspase-3 and caspase-9 in a dose-dependent manner. Additionally, it was observed that GPD-12 downregulated the anti-apoptotic protein Bcl-2 while upregulating the pro-apoptotic protein Bax. Molecular modeling conducted through an in silico approach revealed that among all the derivatives, GPD-12 exhibits the highest interaction with both target proteins, GRP78 and IRE-1. Specifically, GPD-12 interacts with the GRP78 target at Asp257 and Phe114, forming pi–cation and pi–pi stacked interactions, respectively. Additionally, with the IRE1 target, GPD-12 interacts with Ile173 through pi–sigma bond interactions [60].

In a study of 3-*O*-prenyl glycyrrhetinic acid (NPC-402), a derivative of GA, a significant dose-dependent reduction in the viability of B16F10 cells was observed in vitro, while normal HaCaT cells showed no notable cytotoxic effect at concentrations below 50 μM. The IC_50_ values were determined to be 16 ± 0.2 μM for B16F10 cells, 27 ± 1.9 μM for A375 cells, and 33.5 ± 2.3 μM for SKMEL-28 cells. Importantly, GA within the tested concentration range did not exhibit cytotoxic effects, unlike its derivatives. The conducted study demonstrated that treatment with NPC-402 induces an ER stress response in cells, as evidenced by the increased expression of GRP78, which activates the unfolded protein response (UPR) and inhibits protein translation through the GRP78-eIF2α-CHOP signaling pathway. Autophagy induction was observed in melanoma cells treated with NPC-402, supported by enhanced formation of acidic vacuoles, lipidation of LC3I/II, upregulation of Beclin1, and downregulation of the cargo adapter protein SQSTM1/P62. Fluorescence and flow cytometry analyses revealed that this GA derivative reduced the expression of Nrf-2 and modulated downstream redox signaling proteins, such as SOD and catalase, likely due to DNA damage and elevated oxidative stress in melanoma cells. Based on the findings, the pro-apoptotic effects of NPC-402 were associated with changes in mitochondrial membrane potential (ΔΨm), cleavage of PARP-1, activation of caspases, DNA damage, and nuclear fragmentation. Additionally, the translocation of cytochrome C to the cytosol was confirmed using confocal microscopy with MitoTracker. The results also indicated that this derivative decreased the activity of the MAPK signaling axis, thereby promoting increased apoptosis [61].

## 6. Ursane-Type Triterpenoids in Melanoma Treatment—In Vitro Studies

### 6.1. Ursolic Acid (UA)

Ursolic acid (UA), the most studied ursane-type triterpenoid, demonstrates significant potential against various skin cancers, as confirmed by in vitro studies (Table 4). In experiments involving the metastatic melanoma cell line WM-266-4, UA significantly decreased cell proliferation at a concentration of 10 μM. The reductions in cell proliferation activity at 10 μM UA were 25%, 10%, and 5% after 4 h, 24 h, and 48 h, respectively. Furthermore, UA was more effective on the WM-266-4 cell line than BA; however, BA proved to be more effective at lower concentrations than UA. When examining the synergistic effects of UA and OA in both a 1:1 ratio and a 3.5:1 ratio at a concentration of 10 μM, no significant enhancement in effects was observed compared to UA alone, suggesting a lack of substantial interaction [70]. Conversely, in the studies conducted by Soica et al. using the A375 cell line, the Alamar Blue assay demonstrated a synergistic anti-tumor effect of UA and OA (in a ratio of 1:1) within the concentration range of 40–100 μM [71].

Other researchers have also confirmed the cytotoxic effect of UA on the A375 melanoma cell line in a separate study, which demonstrated a dose-dependent response. Notably, this study reported that no changes in proliferation were observed in the normal mesenchymal stem cell line across the entire range of UA concentrations tested (25–100 μM) [72]. In another study, researchers examined the effects of UA on melanoma cell proliferation in the A375 cell line as well as three other lines: MM200, Mel-RM, and Me4405. They noted that UA inhibited melanoma cell proliferation starting at concentrations of 20 μM. Significant cytotoxicity was observed in all cell lines incubated with higher UA concentrations after both 24 and 48 h. The strongest effect of UA was seen in the Me4405 cell line, where it caused 100% inhibition of proliferation at concentrations of 50 μM after 24 h and 40 μM after 48 h. Additionally, the authors demonstrated that UA selectively targets melanoma cells, as normal human fibroblasts exhibited significant resistance to the compound [73]. Harmand et al. evaluated the antiproliferative effect of UA on the M4Beu melanoma cell line using the MTT assay. In their study, the application of UA at concentrations of 15 μM and 20 μM resulted in the inhibition of M4Beu cell proliferation, while a slight increase in proliferation was observed after 24 and 48 h of incubating M4Beu cells with UA at concentrations ranging from 5 μM to 12.5 μM [74]. The inhibitory effect of UA on the proliferation of the M4Beu cell line was also confirmed in the study by Hassan et al., which found that the IC_50_ did not exceed 15 μM. Additionally, the anti-proliferative effect was characterized by a significant proportion of round floating cells in the culture medium, indicative of apoptotic features [75].

In the case of the G361 cell line, UA significantly affected both the number and proliferation of cells, with the most pronounced impact observed at a concentration of 20 µM [76]. Additionally, another study focused on the SK-MEL-2 human melanoma cell line demonstrated that UA exerted an inhibitory and dose-dependent effect on cell growth, yielding an IC_50_ value of 58.43 µM [77].

In normal cells, the cell cycle is carefully regulated. However, in cancer cells, genetic changes disrupt this regulation, leading to uncontrolled growth. It is believed that UA targets cancer cells by arresting specific phases of the cell cycle. In a study conducted by Oprean et al. flow cytometric analysis revealed that UA caused an arrest in the G0/G1 phase in a dose-dependent manner for A375 melanoma cells. The results of annexin V/PI staining, which assessed the apoptotic effect of UA on cells, demonstrated a direct correlation with the concentration to which the cells were exposed. Compared to the control, the population of early apoptotic cells progressively increased, with a difference of up to 36% at the highest concentration tested [72]. In contrast, a previous study by Caunii et al., based on research conducted on the SK-MEL-2 cell line, found that the antiproliferative effects of UA were mediated by changes in the cell cycle, with exposure to 50 µM UA resulting in an arrest in the S phase [77]. In the Me4405 cell line, the involvement of apoptosis in the anti-proliferative effect of UA was confirmed by a significant increase in the DNA content of the sub-G1 peak after 24 and 48 h, which was directly proportional to the concentrations of UA administered [73].

Research suggests that UA can trigger apoptosis in melanoma cells by activating caspase-3. In a study by Paduszyński et al., exposing G361 cells to UA at concentrations of 10 μM and 20 μM resulted in significant caspase-3 activation [76]. Similarly, Mahmoudi et al. reported that UA, at concentrations ranging from 10 to 40 μM, stimulated the proteolytic processing of caspase-3 in Me4405 cells, as confirmed through Western blot analysis [73]. Likewise, Harmand et al. observed caspase-3 activation in M4Beu cells following treatment with UA at 20 μM and 40 μM for 24 h [74]. Duval et al. investigated the impact of UA on key caspase activity using a fluorimetric assay (CaspACE Assay System Fluorometric). When M4Beu melanoma cells were treated with UA at 12.5 µM and 15 µM, caspase-3 activity increased after both 24 h (1.74- and 2.12-fold compared to the control) and 48 h (1.27- and 1.98-fold compared to the control). Additionally, caspase-9 activity showed a slight increase, reaching a maximum of 21% after 48 h [78].

The *bcl-2* gene family encodes a diverse group of proteins that regulate programmed cell death by either promoting or inhibiting apoptosis. Oprean et al. confirmed, through gene expression analysis, that UA significantly induces apoptosis in A375 melanoma cells via the *BCL-2* gene family, resulting in a reduction in *BCL-2* gene expression, while no changes were observed in *BAX* expression [72]. In contrast, other researchers observed that UA altered the BAX–BCL-2 balance, increasing *BAX* expression and decreasing *BCL-2* expression in M4Beu melanoma cells. Additionally, it was observed that UA inhibited the phosphorylation of Akt and ERK-1/2 proteins, resulting in the inactivation of Akt/ERK signaling pathways associated with cell growth and survival, which in turn promoted the induction of apoptosis [75].

Furthermore, the impact of UA was evaluated in B16F-10 mouse melanoma cells. According to Manu et al., in UA-treated cells, the pro-apoptotic genes *P53* and caspase-3 were upregulated, while the anti-apoptotic gene *bcl-2* was downregulated. Additionally, transcription factors *NF-κBp65*, *NF-κBp50*, *NF-κBc-Rel*, *c-FOS*, *ATF-2*, and *CREB-1* were significantly inhibited compared to the untreated control. UA treatment also resulted in the downregulation of pro-inflammatory cytokine production and gene expression of *TNF-α*, *IL-1β*, *IL-6*, and *GM-CSF* in comparison to untreated B16F-10 cells. It was concluded that UA induced apoptosis by inhibiting the NF-κB-induced, bcl-2-mediated anti-apoptotic pathway, while simultaneously activating the p53-mediated and TNF-α-induced, caspase-3-mediated pro-apoptotic pathways [79].

To tackle its low solubility in water, which adversely affects its bioavailability, UA was also investigated in various pharmaceutical formulations aimed at improving these properties. One of the strategies to increase UA water solubility is synthesis of cyclodextrin (CD) inclusion complexes, which add a hydrophilic outer surface. Incorporation of UA in 2-hydroxypropyl-γ-cyclodextrin (HPGCD) in a molar ratio of 1:1 resulted in an increase of the anti-proliferative effect on A375 human melanoma at the concentration of 85µM [71]. Changing the ratio of UA:HPGCD to 1:2 significantly improved the effect on A375 cells, resulting in 96.53% proliferation inhibition at the concentration 75 µM. The complex in the same molar ratio also inhibited the proliferation of SK-MEL-2 cells by 99.48%. UA complexed with 2-hydroxypropyl-γ-cyclodextrin (molar ratio 1:2) was also effective against A375 cells proliferation, reaching the value of 81.94% inhibition at the concentration of 75 µM [80]. Acetylation can change the mechanism behind the anti-proliferative effect of UA on melanoma cells. UA and its derivative, 3-*O*-acetylursolic acid (UAA), both exhibited anti-proliferative properties (GI_50_ = 32.4 µM and 26.7 µM, respectively) on A375 cells, with UAA inducing cell cycle arrest at S phase, while UA at sub-G1 phase. Both agents triggered caspases 3/7 activity, increased Bax levels, and reduced Bcl-2 expression. However, 3-acetylation decreased the anti-migratory effect of UA [81]. DOTA (1,4,7,10-tetraazacyclododecane-1,4,7,10-tetraacetic acid) is widely used in diagnostic imaging but not in the therapy of cancer. However, a piperazine-spacered conjugate of UA and DOTA showed high cytotoxicity against A375 cells (EC_50_ = 1.5 µM). The conjugate worked mainly by apoptosis and inducing cell cycle arrest at the sub-G1 phase [82]. The benzotriazole ester of ursolic acid, 1H-Benzotriazole-1-yl (3β) 3-hydroxyurs-12-en-28-oate, at the concentration of 50 µM reduced cell viability in A375 to 77% and showed no toxicity to HaCaT cells. Pro-apoptotic activities were documented: nucleus shrinkage, membrane fragmentation, reduced Bcl-2 expression, and increase in Bax production [83].

As presented above, UA exhibits a dose-dependent response, inhibiting proliferation in multiple melanoma cell lines, including A375, MM200, Mel-RM, WM-266-4, and Me4405, while sparing normal mesenchymal stem cells. Mechanistically, UA induces apoptosis by activating caspase-3 and promoting DNA fragmentation. Flow cytometric analyses show that UA causes cell cycle arrest, particularly in the G0/G1 phase. Additionally, UA alters the expression of BCL-2 family proteins, reducing BCL-2 levels and increasing BAX levels, which further promotes the apoptosis. Moreover, UA demonstrates anti-inflammatory effects through the downregulation of pro-inflammatory cytokines and inhibition of NF-κB signaling, which enhance the anticancer effect. Overall, UA’s diverse mechanisms underscore its potential as a therapeutic agent for melanoma treatment.

**Table 4:** Ursane-type triterpenoids in melanoma in vitro treatment.

Type	Cell Line	Effect	Concentration/IC_50_ Values	Reference
UA in combination with OA (1:1, 3.5:1)	Human metastatic melanoma cell line (WM-266-4)	↓ cell proliferation activity	0.02, 0.2 μM, and 2 μM	[70]
UA	Human skin melanoma cell lines (A375 and B164A5)	↑ cell cytotoxicity, ↑ apoptosis, ↓ *bcl-2* anti-apoptotic gene expression, arresting cells in the G0/G1 phase	25–100 μM	[72]
UA	Human skin melanoma cell line (SK-MEL-2)	↓ dose-dependent effect on cell growth, cell arrest in the S phase	IC_50_ = 58.43 µM	[77]
UA	Human skin malignant melanoma (G361)	↓ cellular growth, ↑ apoptosis via activation of caspase-3, ↓ DNA synthesis rate	10 μM, 20 μM	[76]
UA	Human skin melanoma cell line (M4Beu)	↓ cell proliferation activity, ↑ apoptosis, ↑ mitochondrial intrinsic pathway, ΔΨm collapse and accumulation of Bax proapoptotic protein, ↑ caspase activation and AIF leakage	5–20 μM	[74]
UA	Human melanoma cells (M4Beu), normal human keratinocytes (HaCaT)	↑ significant caspase-3 activation, ↑ strong ΔΨm collapse in cancer cells, change in Bax/Bcl-2-balance in favor of Bax	12.5 μM, 15 μM	[78]
UA	Human melanoma cell lines (MM200, Mel-RM, Me4405, and A375)	↓ cell proliferation after 24 and 48 h, ↑ proteolytic processing of caspase-3	10–40 μM	[73]
UA	Human melanoma cells (M4Beu)	↓ phosphorylation of Akt and ERK-1/2 proteins, inactivation of cell growth and survival-related Akt/ERK signaling pathways, ↑ apoptosis induction	10–17.5 μM	[75]
UA	Human melanoma cell line (A2058)	↑ apoptosis	50–75 μM	[72]
UA; inclusion complex of UA and 2-hydroxypropyl-β-cyclodextrin (UA: HPβCD) in the molar ratio of 1:2; inclusion complex of UA and 2- hydroxypropyl-γ-cyclodextrin (UA: HPγCD) in the molar ratio of 1:2	Human melanoma cell line (A375 and SK-MEL-2)	↓ cell proliferation	A375: 5–50 µM, UA: IC_50_ = 68.22 µM, (UA: HPβCD): IC_50_ = 51.73 µM, (UA: HPγCD): IC_50_ = 31.38 µM, SK-MEL-2: 5–50 µM, UA: IC_50_ = 58.44 µM, (UA: HPγCD): IC_50_ = 9.26 µM	[80]
UA; UAA	Human melanoma cell line (A375)	↓ cell proliferation, ↑ caspases 3/7 activity, ↑ Bax levels, ↓ Bcl-2 production, cell cycle arrest at sub-G1 phase (UA) or S phase (UAA)	GI_50_ = 32.4 µM, GI_50_ = 26.7 µM (respectively)	[81]
Piperazine-spacered conjugate of ursolic acid and 1,4,7,10-tetraazacyclododecane-1,4,7,10-tetraacetic acid	Human melanoma cell line (A375)	↑ apoptosis, ↑ number of cells in sub-G1 phase	EC_50_ = 1.5 µM	[82]
1H-Benzotriazole-1-yl (3β) 3-hydroxyurs-12-en-28-oate	Human melanoma cell line (A375)	↓ cell viability, ↑ apoptotic activities (nucleus shrinkage, ↓ Bcl-2 expression, ↑ Bax production	50 µM	[83]
AA	Human melanoma cell line (SK-MEL-2)	↓ cell viability, ↑ apoptosis in a dose-dependent manner, ↑ intracellular ROS, ↑ expression of Bax, ↑ activation of caspase-3	40 μM	[84]
AA derivative	Human melanoma cell line (A375)	↑ cell cytotoxicity	0.0028, 0.58, 1.3, or 30 μM	[85]
MA; MA homopiperazinyl rhodamine B conjugate	Human melanoma cell line (A375)	no significant effect (MA), strong cytotoxic effect (conjugate)	EC_50_ > 30 µM, EC_50_ = 0.0095 µM (respectively)	[86]
Methyl 2-oxo-3β-(2-furoyloxy)-6,23-epoxyursa-5,12-dien-28-oate (MA derivative)	Human melanoma cell lines (SK-MEL-5, UACC-257)	↓ cell growth	TGI = 2.6 µM, LC_50_ > 100 µM, TGI = 3.0 µM, LC_50_ = 50.1 µM (respectively)	[87]
α-amyrin	Human melanoma cell line (A375)	↓ cell proliferation activity	IC_50_ = 1.26 µM	[54]

UA—ursolic acid, UAA—3-O-acetylursolic acid, AA—asiatic acid, MA—madecassic acid, ↓—decreased/alleviated, ↑—increased/induced.

### 6.2. Asiatic Acid (AA)

Asiatic acid (AA), a pentacyclic triterpene acid from the extracts of *Centella asiatica*, effectively induced apoptosis in human SK-MEL-2 melanoma cells, with an IC_50_ of approximately 40 μM. Early-stage apoptosis studies showed that AA initiates the loss of lipid asymmetry, leading to the exposure of phosphatidylserine on the outer layer of the plasma membrane and inducing apoptosis in a dose-dependent manner. The studies demonstrated that the apoptosis induced by AA occurs through the generation of ROS, alterations in the Bax/Bcl-2 ratio, and activation of caspase-3 in SK-MEL-2 human melanoma cells, without any changes in p53 protein levels [84].

Recently, in the A375 cell line, newly synthesized rhodamine B hybrids derived from AA demonstrated significant cytotoxicity. The SRB assay revealed that after 72 h of treatment, the EC_50_ value for the melanoma cell line ranged from 0.0028 to 30 μM, with the activity of these conjugates influenced by the size of the spacer connected to the carboxyl group in ring E. To quantify the ratio of apoptosis to necrosis, the annexin V/propidium iodide (A-V/PI) method was utilized. Treatment of A375 cells with rhodamine B conjugate of AA for 24 h resulted in 81.4% of the cells remaining viable; after 2 days, 44.1% of the cells were apoptotic, while only 3.9% were necrotic. These results clearly indicate that A375 cells predominantly undergo apoptosis rather than necrosis [85].

### 6.3. Madecassic Acid (MA)

MA, next to AA, is another aglycone isolated from *Centella* spp. Only a small number of MA derivatives have been identified, and few of them have been studied for their cytotoxic properties. Out of 29 derivatives tested by Valdeira et al. [87], methyl 2-oxo-3*β*-(2-furoyloxy)-6,23-epoxyursa-5,12-dien-28-oate showed the best ability to totally inhibit the growth of SK-MEL-5 and UACC-257 melanoma cells (TGI = 2.6–3.0 µM). However, this compound did not exhibit the strong cytotoxic effect of the latter cell lines (LC_50_ > 50 µM). An MA homopiperazinyl rhodamine B conjugate acted as mitocan and exhibited a strong cytotoxic effect on A375 melanoma cells (EC_50_ = 0.0095 µM), which was several thousand times stronger than that from the parent compound (EC_50_ > 30 µM). The conjugate also turned out to be more selective towards cancer cell lines than MA [86].

### 6.4. α-Amyrin

Amyrins can be obtained from different plant materials, mostly from cuticular waxes. *α*-Amyrin is an ursane, while *β*-Amyrin is an oleanane triterpenoid. They frequently occur together [88]. α-Amyrin was reported to have a strong cytotoxic effect (IC_50_ = 1.26 µM) on A375 melanoma cells, surpassing that of cisplatin (IC_50_ = 2.06 µM). However, its low water solubility hinders its use in pharmaceuticals [54].

## 7. Triterpenoids in Melanoma Treatment—In Vivo Studies

Despite the multitude of in vitro studies on the impact of different types of terpenoids on skin cancer, the number of tests performed on laboratory animals is still scarce. However, as visualized by the scientific databases (e.g., by the Scopus database), the last 6 years bring an increasing number of publications that undertake the topic of skin cancer treatment with the help of terpenoids.

Among the representatives of lupane, oleanane, and ursane types of saponins, oleanolic and ursolic acids have certainly been studied the most thoroughly for their anticancer potential among the known terpenoids and for their impact on skin tumors.

In the study of Caunii et al., the influence of OA and UA on normal and tumor angiogenesis in melanoma was performed using the chicken embryo vascular membrane (CAM) assay. The experiment included a study on 7–11-day-old embryos that were inoculated with SK-MEL-2 melanoma cells. The tested groups were treated with 30 mM concentrations of either UA or OA (5-μL volumes) for 5 days, starting on the first day after inoculation of the embryos with SK-MEL-2 cells. The study showed that both OA and UA caused a reduction in the number of blood vessels in the embryos at the application sites of both solutions, with a more pronounced anti-angiogenic effect observed for OA. OA not only reduced the density of blood vessels but also induced changes in the arrangement of vessels surrounding tumor cells, which led to the slower growth of the tumor. On the other hand, UA did not significantly alter the capillary density, and the invasiveness of melanoma in the UA group was higher than in the OA-treated one. Nevertheless, the results of this study showed that neither acid inhibited the metastatic potential of SK-MEL-2 cells [77].

However, OA was found to reduce metastasis in another model of melanoma, assessing the condition of xenograft 10–12-week-old C57BL/6 mice with B16F10 cells injected subcutaneously. The treatment with 20 μL of 10 mg/kg b.w. solution of OA started three days after inoculation with melanoma cells and lasted for 2 consecutive weeks (a single dose administered five times a week). It was noted that within the groups treated with OA solution, the number of metastatic tumor cells was significantly lower compared to the control and DMSO groups. Thus, the study showed that OA was responsible for inhibiting melanoma cell proliferation [89].

The assessment of the chemopreventive effects of pure OA and UA and the same components encapsulated in polyurethane (PU) nanostructures was the aim of another study. The scientists used 12-week-old female SKH1 mice treated with 200 μL of the tumor initiator DMBA (7,12-dimethylbenzanthracene) applied once a week for a fortnight to the dorsal skin of the animals to determine the anticancer effects of terpenoids. In the third week, the mice were divided into five groups and treated with 200 μL of the tumor promoter TPA (12-tetradedecumiforbol) twice a week for 29 weeks. Thirty minutes prior to the application of TPA, the mice were treated according to the group to which they belonged: with 200 μL 10μM UA solution, 200 μL 10μM PU-UA complex solution, 200 μL 10μM OA solution, 200 μL 10μM PU-OA complex solution, or with saline (control group). All groups developed papilloma after exposure to DMBA and TPA. The experiment showed that the UA and PU-OA groups had a reduced incidence of cancer compared to the other groups. The number of tumors decreased in the UA, PU-UA, PU-OA groups. Intriguingly, there was an increase in papilloma in the UA group, while PU-UA developed keratinizing squamous cell carcinoma despite the reduction in tumor numbers. It was concluded that UA gives moderate skin protection and that polyurethane structures are not a good carrier for OA and UA [90].

The anticancer properties of OA were supplemented by the studies of Woo and co-investigators, who performed studies on xenografts with A375SM cells injected into the shoulders of 4-week-old female BALB/c nude mice. The treatment with a daily dose of 75 or 150 mg/kg b.w. of OA (*n* = 5) for 13 consecutive days showed a significant reduction in the tumor size and volume, especially in the group treated with a higher dose of OA. Additionally, an elevated number of TUNEL-positive cells was noted, which suggested the impact of OA on apoptosis in melanoma cells. Further studies on the impact of terpenoids on skin cancer also engage other molecules, including AA and BA, standing for the anticancer potential of this group of secondary metabolites. In recent studies AA was tested for its anticancer potential towards skin carcinoma in a similar mice model using DMBA and TPA for the promotion of tumor growth in 6-week-old female ICR rodents. A 20-week-long treatment with two doses of AA, namely 30 or 50 μmol, of the terpenoid 1 h before the administration of TPA twice a week resulted in fewer tumors. The average number of tumors in the control group was calculated as eight, while both treated groups showed only five tumors on average. The incidence of tumors because of the study was only diminished but not totally inhibited after all. The potential mechanisms of action that were revealed for the AA were the ability to inhibit TPA-induced NO production and the expression of iNOS and cyclooxygenase-2 (COX-2), which play important roles in tumor growth [48].

Promising antitumor effects were observed in the combined therapy of melanoma using AA and naringenin (NG). In the study, 8-week-old male C51BL/6 mice injected with B16F10 tumor cells were treated either with AA (10 mg/kg body weight) or NG (5 g/kg body weight) alone or with both drugs (AA-NG, 10 mg/kg + 50 mg/kg). The researchers underlined that restoring a balance between Smad 3 and Smad 7 signaling, which occurred by using AA, a Smad 7 inducer (restored expression), and NG, a Smad3 inhibitor (inhibited translation and phosphorylation), led to the alleviation of tumor progression in vivo. The proposed therapy induced the differentiation and maturation of NK cells supporting the anti-tumor action. The combined treatment elevated the NK cell immunity against cancer by employing the Id2- and IRE2-based mechanisms, which resulted in a higher cytotoxicity. Also, as shown by the authors, the AA-NG combination reduced melanoma volume compared to AA monotherapy and did not alter leukocyte counts standing for lack of nephro-, cardio-, or hepatotoxic effects. Based on the results obtained, it can be concluded that the indicated additive effect of NG and AA may be worth considering for a promising immunotherapy of melanoma patients [91].

BA was proved to reduce melanoma proliferation, tumor weight, and development in an experiment on 8-week-old C57BL/6J mice divided into two groups: those inoculated with B164A5 melanoma cells (control) and those inoculated with B164A5 melanoma cells and treated with BA solution at the concentration of 100 mg/kg i.p. every day for 21 days. Additionally, a reduction in melanin clustering and erythema was observed, which is particularly important given that the B164A5 line leads to skin hyperpigmentation. In addition, the authors observed a reduction in hemoglobin levels, suggesting that BA may have an anti-angiogenic effect [92].

In another trial, BA exerted a potentiating effect on the anticancer action of vincristine (VCR) when tested in mouse xenografts with metastatic melanoma (B16F10 cells). The study used 9–10-week-old female C57BL/6 mice that were injected with B16F10 melanoma cells into their veins. The animals were divided into four experimental groups: a control group and groups treated with (1) 10 mg/kg BA solution, (2) 0.065 mg/kg VCR solution, and (3) 10 mg/kg BA and 0.065 mg/kg VCR solutions. Upon the treatment, tumors arising within the metastases were measured, and it was noted that the control group developed nodules of 6.0 ± 12.2 mm in size, while the other groups had values of 14.0 ± 2.0, 16.8 ± 9.0, and 3.6 ± 5.5 mm, respectively. The ratios of the number of large nodules to the total number of nodules were also determined and were 71.7 for the control group and 50.0, 47.2, and 29.0%, respectively, for the treated groups. The results show that both VCR and BA suppress the growth of B16F10 cells. Furthermore, the greatest response was shown by group 3, indicating that BA enhances the effect of VCR, and this suggests that the VCR-BA complex may be a promising therapy in metastatic melanoma [93].

Terpenoids were also found to be helpful in the treatment of non-melanoma skin cancers (NMSCs), which are the most common cancers that occur because of ultraviolet irradiation. The beneficial action of UA was determined in 8-week-old SKH-1 hairless mice with ultraviolet B ray-induced carcinoma. In this model, the tumors were formed by irradiating the animals’ skin with UVB rays. In the same study, the animals were divided into three groups, one of which combined the irradiation with the treatment with UA solution. After 25 weeks, the skin samples of the control and UA-treated groups were collected post-mortem for the sequencing studies and subjected to the identification of genes that were modified both by the tumor and by the treatment with UA. A significant reduction in tumor volume and number was observed upon treatment with 2 µM UA solution (200 µL). Next to these effects, it was noted that UA activated the Nrf2-mediated response to oxidative stress, especially at the initiation stage, but during the tumor promotion and progression, the mechanism of action was opposite. Also, UA increased the activity of the IL-8 signaling pathway at the beginning of tumor growth and alleviated its activity at the more advanced stages of tumor development. The authors do not exclude the potential modulatory impact of UA on antioxidative and inflammatory pathways such as Nrf2 (Table 5) [94].

Terpenoids have garnered significant attention for their potential applications in skin cancer treatment, particularly due to their diverse mechanisms of action. Despite limited studies on laboratory animals, recent years have seen an increase in research focusing on terpenoids like OA and UA, which have shown promising anticancer effects. In the comparative experiments both OA and UA displayed anti-angiogenic properties, with OA demonstrating a more pronounced effect by reducing blood vessel density and altering vessel arrangement, thus inhibiting tumor growth.

Moreover, OA has been shown to significantly reduce metastasis in mouse models, highlighting its potential in combating melanoma spread. Studies have also revealed that UA exhibits chemopreventive effects, reducing tumor incidence when encapsulated in nanostructures. Noteworthy, the combinations of UA and naringenin or BA and vincristine have been linked to enhanced immune responses against tumors, presenting a potential avenue for an efficient immuno- and chemotherapy. Furthermore, terpenoids have also been effective against non-melanoma skin cancers, with UA demonstrating protective effects against UV-induced carcinogenesis. Overall, the emerging evidence underscores the significance of terpenoids in practical therapeutic applications for skin cancer, warranting further exploration and development.

**Table 5:** The effects of triterpenoid treatment on skin cancer reported from the in vivo experiments.

Compound	Organism	Model	Duration and Dose	Effect and Mechanism	Reference
AA	6-week-old female ICR mice	Xenografts with tumor initiator (DMBA) and tumor promoter (TPA)	20 weeks, two doses of AA (30 and 50 μM) twice a week 1 h before TPA	↓ average number of tumors, ↓ TPA-induced NO production, ↓ expression of NO synthase (iNOS) and cyclooxygenase-2 (COX-2)	[95]
AA and naringin	8-week-old male C51BL/6 mice	Xenografts with melanoma cells (B16F10 tumor cells)	Daily dosing, AA (10 mg/kg body weight) or NG (50 mg/kg body weight) alone or with both drugs (AA-NG, 10 mg/kg + 50 mg/kg)	↓ volume of melanoma in the joined therapy, no cardio-, nephron or hepatotoxic effects, ↑ maturation and differentiation of NK cells, ↑ NK cells immunity against cancer cells (via Id2 and IRE2 mechanisms), ↑ Smad 7 expression, ↓ Smad 3 translation and phosphorilation	[91]
BA	8-week-old C57BL/6J mice	Xenografts with melanoma cells (B164A5 tumor cells)	Daily for 21 days, 100 mg/kg i.p. BA	↓ hyperpigmentation, ↓ erythrema, ↓ angiogenesis, ↓ proliferation, tumor size, and weight	[92]
BA	9–10-week-old female C57BL/6 mice	Mouse xenografts with metastatic melanoma (B16F10 cells)	10 mg/kg b.w. BA solution; 0.065 mg/kg b.w. vincristine; a combination of both	In the combined therapy: ↓ total number of nodules, ↓ the ratio of the total number of large to small nodules	[93]
OA	C57BL/6 mice	Xenografts with melanoma cells	Ten 20 μL doses of 10 mg/mL solution of oleanolic acid within 2 weeks	↓ melanoma cell proliferation	[89]
OA	4-week-old female BALB/c nude mice	Xenografts with melanoma (A375SM cells)	75 mg/kg and 150 mg/kg OA solution (n = 5), five times a week for 13 days	↑ apoptosis, ↑ number of TUNEL-positive cells, -> NF-ĸB-mediated anticancer effects	[48]
UA	8-week-old SKH-1 hairless mice	Irradiation with UVB for 25 weeks with and without UA	UVB with 2 µM UA solution (200 µL)	↑ IL-8 signaling pathway, ↑ Nrf2-mediated response to oxidative stress, ↓ tumor size and number	[94]
UA and OA	Chicken embryos (7–11 day old) inoculated with SK-MEL-2 melanoma cells	Vascular membrane assay	Five daily doses of 30 mM of each acid	↓ number of vessels (stronger in the OA group), ↓ invasiveness of melanoma in OA-treated group, ↑ changes in the arrangement of vessels surrounding tumor cells	[77]
UA, OA, and loaded with UA and OA polyurethane nanoparticles	12-week-old female SKH1 mice	Xenografts with tumor initiator (DMBA) and tumor promoter (TPA)	29-weeks, 200 μL 10μM of UA, OA, PU-UA, or PU-OA solutions twice a week	↓ incidence of cancer in UA and PU-OA groups, ↓ number of tumors in UA, PU-UA, PU-OA groups, ↑ increased papilloma in keratinized, ↑ squamous cell carcinoma in PU-UA	[90]

AA—asiatic acid, BA—betulinic acid, OA—oleanolic acid, UA—ursolic acid, ↓—decreased/alleviated, ↑—increased/induced.

## 8. Discussion and Prospects

Lupane-, oleanane- and ursane-type triterpenoids have garnered attention for their potential in melanoma treatment, exhibiting various anticancer properties. Compounds such as BA, UA, and OA have demonstrated cytotoxic, antiproliferative, and pro-apoptotic effects against melanoma cells in vitro and in vivo. Currently, based on the available scientific evidence, terpenoids can be considered moderately active inhibitors of skin cancer. Several of the aforementioned studies demonstrate their potential by indicating a tendency to suppress tumor formation, inhibit angiogenesis, and modulate inflammatory mediators such as COX-2 or NO, which are involved in tumor progression.

Terpenoids possess significant potential for incorporation into therapeutic practices, not only due to their proven anticancer properties in the treatment of skin cancers but also because of their considerable safety profile. Several studies on the toxicity associated with various terpenoids, specifically OA, UA, BA, hederagenin, and α- and β-amyrin, have been published in scientific publications. The findings revealed that these compounds exhibited significant cytotoxic effects on melanoma cells, but their toxicity towards healthy cells and vital organs remained relatively low. An oral acute toxicity assessment of OA and UA in a rat model was performed, during which no pathological alterations were detected in major organs, including the kidneys, liver, brain, spleen, uterus, and heart. Furthermore, there were no observed changes in animal behavior [96]. A study led by Huang determined that the median lethal dose (LD50) of OA was 173.3 mg/kg body weight [97]. Also, Mishra et al. thoroughly investigated the oral acute and subacute toxicity of BA and UA. Their research indicated that BA induced a transient increase in the serum levels of SGOT (serum glutamate oxaloacetic transaminase), alkaline phosphatase (ALP), and urea; however, these levels normalized following the cessation of treatment. Importantly, no significant alterations in myocardial structure were observed. The researchers concluded that the LD50 of BA exceeded 2000 mg/kg body weight, suggesting a low toxicity profile [98]. Interestingly, another investigation into escin revealed that oral administration at a dose of 300 mg/kg body weight resulted in no observable toxicity, whereas a dose of 2000 mg/kg body weight proved lethal in rats. At lower doses of 5, 10, and 20 mg/kg/day, no significant changes in food and fluid intake or body weight were noted among the animals [99]. Concerning hederagenin, a moderate cytotoxicity at higher concentrations (above 100 µg/mL) in mouse fibroblasts was observed, while lower concentrations (below 50 µg/mL) were well tolerated by the cells [100]. In the case of toxicity evaluation of α- and β-amyrin, in a study with oral administration of up to 2000 mg/kg body weight, no mortality was observed. Moreover, no significant alterations in hematological or biochemical parameters were noted at lower doses. Symptoms of physical fatigue and weakness were only observed at doses greater than or equal to 1000 mg/kg, leaving a broad safety margin [101]. Notably, a literature review yielded no data regarding the toxicity of GA and MA, nor were there findings related to the long-term effects of terpenoids on the toxicity of healthy cells. Consequently, it is imperative that further detailed studies are conducted to unequivocally establish toxic doses of terpenoids on healthy cells and to ascertain safe concentration levels for both humans and animals.

However, despite these promising properties, especially of lupane-, oleanane-, and ursane-type triterpenoids, their efficacy in tumor treatment at the tested doses is often limited, resulting in only partial tumor regression or a significant yet insufficient reduction in tumor burden. The clinical application of these triterpenoids faces challenges primarily due to their limited bioavailability and solubility. That is why comprehensive clinical evaluations and advanced formulation strategies are essential to fully harness their therapeutic benefits.

Addressing these issues, implementing nanoformulations as tools for transporting therapeutically active compounds represents a crucial direction in drug formulation development, as it leads to the formation of recent research that has focused on developing novel pharmaceutical formulations, such as nanoformulations and derivatives with improved pharmacokinetic profiles, to enhance the therapeutic efficacy of natural products. These studies also relate to terpenoids’ applications in melanoma treatment. As proved by Ghiulai et al., gold nanoparticles served as versatile carriers for pentacyclic triterpenes, enhancing the bioavailability and therapeutic efficacy of the lipophilic structures. An in vitro biological assessment of BA-functionalized GNP, synthesized by grafting BA onto citrate-capped GNP using cysteamine as a linker, was performed in HaCaT human keratinocytes and RPMI-7951 human melanoma cells, demonstrating selective cytotoxicity and enhanced antiproliferative effects compared to free BA. Additional investigations revealed a pro-apoptotic effect, evidenced by morphological changes in melanoma cells, showing the downregulation of anti-apoptotic Bcl-2 and upregulation of pro-apoptotic Bax. Moreover, GNP significantly inhibited mitochondrial respiration, indicating its targeted activity on the mitochondria [43].

Additionally, as demonstrated above, combination therapies involving triterpenoids and other anticancer agents or physicochemical factors have shown potential in enhancing treatment outcomes. The growth-inhibitory properties of BA, both alone and in combination with ionizing radiation, across various established human melanoma cell lines and normal human melanocytes brought positive results. According to the study, BA effectively and consistently suppressed the growth and colony-forming ability of all melanoma cell lines examined, and—when combined with ionizing radiation—the growth-inhibitory effect of BA was additive in colony-forming assays. The induced apoptosis was evidenced by annexin V binding and an increased expression of the Mcl-1 protein in cancer cells versus normal melanocytes, suggesting that BA is a promising candidate for use as a monotherapy and in combination with radiotherapy [102]. Also, combinations with other drugs seem to be promising for terpenoids and melanoma treatment. BA—the most studied terpenoid—substantially inhibited the growth of melanoma cell lines in vitro, with the IC_50_ values ranging from 2.21 µM to 15.94 µM across the four lines tested. Furthermore, co-treatment with BA and either paclitaxel or docetaxel resulted in favorable drug–drug interactions, characterized by additive effects and a tendency toward synergy, which was proved by the isobolographic analysis. Notably, BA exhibited no cytotoxic effects on normal human keratinocyte HaCaT cells, with significantly lower levels of LDH release compared to melanoma cell lines [44]. Unfortunately, the number of studies investigating these combinations remains limited, and further research is necessary to establish their efficacy and safety profiles. An important issue for terpenoid-based treatment is their chemical character. Taking advantage of their physicochemical properties may lead to the introduction of more efficient therapeutic strategies.

Terpenoids play a significant role in the organization of biological membranes across all forms of cellular life on Earth. Notably, polycyclic terpenoids, including hopanoids and steroids, serve as permanent membrane regulators in both bacteria and eukaryotes. Additionally, carotenoids, quinones, and polyprenoids are primarily associated with photosynthesis, respiration, and cell envelope biogenesis, respectively, while also influencing the properties of surrounding membrane environments [103]. A deeper insight into the membranotropic characteristics of triterpenoids would be beneficial. These compounds can modify membrane dynamics via affecting the function of membrane-associated proteins, such as those in the mitochondrial electron transport chain, as well as membrane permeability. Novel cationic dimethylaminopyridine (DMAP) derivatives of pentacyclic triterpenes have been reported to induce mitochondrial depolarization and cell death in breast and melanoma cell lines, which can constitute an important mechanism of action in the treatment of carcinoma. The study of Serafim et al. revealed a significant inhibition of cell proliferation, accompanied by G1-phase cell cycle arrest, notable loss of mitochondrial membrane potential, and further activation of caspase-9 and caspase-3, leading to cell death, especially in galactose-containing media that was induced by the DMAP derivatives. To provide direct evidence, isolated rat liver mitochondria were utilized, demonstrating that the compounds are strong inducers of the permeability transition pore. These results confirm previous findings, indicating that the novel DMAP derivatives interact robustly with mitochondria, triggering pro-apoptotic signaling and subsequent cell death [104]. Gaining a deeper understanding of these mechanisms would extend the relevance of anticancer research on terpenoids, as mitochondrial targeting and membranotropic effects are universal strategies that can be applied both to melanoma and various other cancers.

The newest approach towards anticancer treatment is related to the study of mitochondria-targeted drugs. Mitochondria are universal targets for anticancer agents as they are presence in the majority, if not all, types of transformed cells. They are characterized by having critical roles in energy production, regulation of apoptotic pathways, generation of ROS, and maintenance of calcium homeostasis. Consequently, mitochondriotropic anticancer agents, or mitocans, that induce mitochondrial destabilization show considerable promise in cancer therapy. As proved by several studies, natural pentacyclic triterpenoids are recognized as promising scaffolds for the development of new mitochondria-targeted anticancer agents. These secondary metabolites interact with the mitochondria inside the tumor cells and trigger the formation of reactive oxygen species, as proved by Spivak et al. [105], who showed the cytotoxic activity of conjugates formed by pentacyclic triterpenoids and various mitochondria-targeted cationic lipophilic molecules. It is worth noting that the anticancer potential of terpenoids has different cellular directions. However, their cytotoxicity is directly linked to their influence on the mitochondria of cancer cells. During the occurring interactions, terpenoids affect the respiratory chain and induce mitochondrial permeability transition (MPT), succeeding with the induction of apoptosis, e.g., through the release of cytochrome c.

It is important to emphasize that the number of studies investigating the anticancer potential of terpenoids remains relatively limited, which makes it challenging to draw definitive conclusions at this stage. Despite this limitation, emerging scientific evidence supports their high safety profile, indicating that terpenoids exhibit low toxicity and are well tolerated in various biological systems. Furthermore, a growing body of research confirms their anticancer properties, revealing mechanisms through which terpenoids can inhibit tumor growth, induce apoptosis, and reduce metastasis in various cancer types.

In addition to their direct anticancer effects, terpenoids have been shown to exhibit several supplementary beneficial activities. These include immunomodulatory effects, where terpenoids can enhance or regulate the immune response, potentially improving the body’s ability to combat cancer. Their antioxidant action helps to neutralize free radicals, thereby reducing oxidative stress, which is often implicated in cancer progression. Additionally, the anti-inflammatory properties of terpenoids may play a crucial role in mitigating chronic inflammation, a known risk factor for various cancers.

Given these diverse attributes, terpenoids present a promising group of compounds for therapeutic applications. They may be utilized not only in monotherapy but also in combination with other therapeutics and treatment modalities, suggesting a synergistic potential that could enhance overall treatment efficacy. This versatility makes them applicable in both traditional herbal medicine and modern pharmaceutical formulations, broadening their potential use in clinical practice.

Collectively, these findings provide a solid foundation for the development of further in vivo studies and clinical trials.

## 9. Conclusions

Terpenoids have demonstrated significant anticancer potential in melanoma through their diverse mechanisms of action. These natural compounds target key signaling pathways involved in tumor progression and drug resistance, making them promising candidates for melanoma therapy. Additionally, some terpenoids enhance the efficacy of conventional treatments, offering potential combination strategies to improve therapeutic outcomes.

As demonstrated in the manuscript, terpenoids often exhibit selective toxicity, effectively targeting cancer cells while sparing normal cells. Due to their low toxicity, terpenoids may be suitable for long-term use in cancer management. This selectivity could result in fewer side effects compared to conventional chemotherapeutic agents, which are commonly associated with significant damage to healthy tissues. Additionally, the anti-inflammatory and antioxidant properties of terpenoids may contribute to reducing the risk of cancer progression. Furthermore, their immunomodulatory effects may enhance the immune system’s ability to recognize and eliminate cancer cells.

Despite their promising preclinical findings, the clinical translation of terpenoids remains challenging due to limitations in bioavailability, stability, and targeted delivery. Advances in nanotechnology and novel drug formulations are addressing these issues, improving the pharmacokinetic properties and therapeutic effectiveness of terpenoid-based treatments.

Future research should focus on optimizing terpenoid formulations, exploring synergistic combinations with existing therapies, and conducting well-designed clinical trials to validate their efficacy and safety. If these challenges are overcome, terpenoids could emerge as valuable therapeutic agents in the fight against melanoma, offering new hope for improved patient outcomes.

## Figures and Tables

**Table 1:** The classification and examples of terpenoid compounds.

Group of Terpenoids	Example of Compound	Plant Sources	Reference
Monoterpenoids	Linalol	*Lavandula angustifolia*, *Citrus bergamia*	[17]
	Thymol	*Thymus vulgaris*, *Origanum vulgare*	[17]
	Thujone	*Artemisia vulgaris*, *Salvia officinalis*	[17]
Diterpenoids	Abietic acid	*Pinus palustris*	[18]
	Forskolin	*Coleus forskohlii*	[19]
Triterpenoids	Oleanolic acid	*Olea europea*	[20]
	Betulinic acid	*Betula pendula*	[21]
	Ursolic acid	*Sambucus nigra*	[20]
	Asiatic acid	*Centella asiatica*	[22]
Tetraterpenoids	Β-carotene	*Solanum lycopersicum*	[23]
	Lutein	*Citrus sinensis*	[23]
Sesquiterpenoids	Cnicin	*Cnicus benedictus*	[24]
	Santonin	*Artemisia annua*	[25]

## Abbreviations

AKT	protein kinase B
ALP	alkaline phosphatase
APC	antigen-presenting cell
CAM	chicken embryo vascular membrane
CD	cyclodextrin
COX-2	cyclooxygenase-2
CTLA-4	cytotoxic T-lymphocyte associated protein 4
DCT	dopachrome tautomerase
DMAP	dimethylaminopyridine
DMAPP	dimethylallyl diphosphate
DMBA	7,12-dimethylbenzanthracene
EGFR	epidermal growth factor receptor
ERK 1/2	extracellular signal-regulated kinases 1/2
GPCRs	G protein-coupled receptors
GTP	guanosine-5′-triphosphate
HO-1	heme-oxygenase 1
HPGCD	2-hydroxypropyl-γ-cyclodextrin
INOS	inducible nitric oxide synthase
IPP	isopentenyl diphosphate
LDH	lactate dehydrogenase
LD50	lethal dose 50
MAPK	mitogen-activated protein kinase
MEB	2-C-methyl-D-erythritol 4-phosphate
MEK 1/2	mitogen-activated protein kinase kinase 1/2
MITF	microphthalmia-associated transcription factor
mTOR	mechanistic target of rapamycin kinase
MTT	3-(4,5-dimethylthiazol-2-yl)-2,5-diphenyltetrazolium
MPT	mitochondrial permeability transition
MVA	mevalonate
NF-κB	nuclear factor-kappa B
NG	naringenin
NMSCs	non-melanoma skin cancers
NO	nitric oxide
OA	oleanolic acid
PD-1	programmed cell death protein 1
PDK1	phosphoinositide-dependent kinase-1
PD-L1	programmed cell death ligand
p-ERK	phosphorylated extracellular signal-regulated kinase
PI3K	phosphoinositide 3-kinase
PIP2	phosphatidylinositol-4,5-bisphosphate
PIP3	phosphatidylinositol-3,4,5-trisphosphate
*p*-IκBα	phospho-inhibitor of nuclear factor-κBα
p–NF–κB	phospho-nuclear factor-κB
PTEN	phosphatase and tensin homolog
PU	polyurethane
ROS	reactive form of oxygen
RTK	receptor tyrosine kinases
SGOT	serum glutamicoxaloacetic transaminase
tBHP	tert-butyl hydroperoxide
TGFβ	transforming growth factor-beta
TIMP-1 and TIMP-2	tissue inhibitors of metalloproteinases 1 and 2
TPA	12-tetradedecumiforbol
TYR	tyrosinase
TYRP1	tyrosinase-related protein 1
UA	ursolic acid
UAA	3-O-acetylursolic acid
UV	ultraviolet
VCR	vincristine
